# Computational modelling of bovine ovarian follicle development

**DOI:** 10.1186/1752-0509-7-60

**Published:** 2013-07-15

**Authors:** Dagmar Iber, Christian De Geyter

**Affiliations:** 1 Department for Biosystems Science and Engineering (D-BSSE), ETH Zurich, Swiss Institute of Bioinformatics, Basel, Switzerland; 2 Division of Gynecological Endocrinology and Reproductive Medicine, Women’s Hospital, University of Basel, Basel, Switzerland

**Keywords:** Ovarian follicle development, PDE model, Computational biology, Bovine

## Abstract

**Background:**

The development of ovarian follicles hinges on the timely exposure to the appropriate combination of hormones. Follicle stimulating hormone (FSH) and luteinizing hormone (LH) are both produced in the pituitary gland and are transported via the blood circulation to the thecal layer surrounding the follicle. From there both hormones are transported into the follicle by diffusion. FSH-receptors are expressed mainly in the granulosa while LH-receptors are expressed in a gradient with highest expression in the theca. How this spatial organization is achieved is not known. Equally it is not understood whether LH and FSH trigger distinct signalling programs or whether the distinct spatial localization of their G-protein coupled receptors is sufficient to convey their distinct biological function.

**Results:**

We have developed a data-based computational model of the spatio-temporal signalling processes within the follicle and (i) predict that FSH and LH form a gradient inside the follicle, (ii) show that the spatial distribution of FSH- and LH-receptors can arise from the well known regulatory interactions, and (iii) find that the differential activity of FSH and LH may well result from the distinct spatial localisation of their receptors, even when both receptors respond with the same intracellular signalling cascade to their ligand.

**Conclusion:**

The model integrates the large amount of published data into a consistent framework that can now be used to better understand how observed defects translate into failed follicle maturation.

## Background

**Figure 1:**
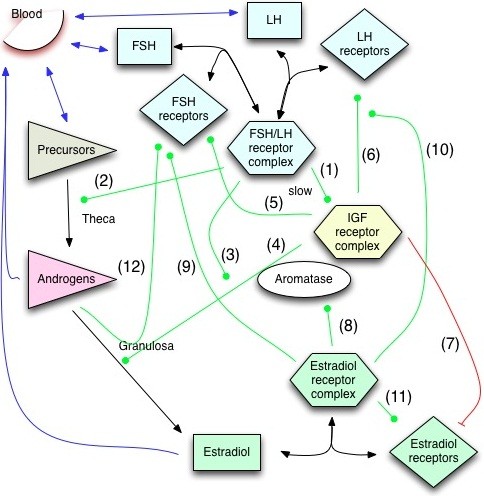
**Regulation of follicular development.** The modelled signalling network. In brief, FSH- and LH-signalling enables IGF signalling in several ways (arrow A1), and enhances the production of androgens (A2), as well as the production and activity of aromatase (A3), the enzyme that catalyzes androgens into estradiol. IGF signalling is necessary for the gonadotropin-dependent expression of aromatase (A4), enhances the production of FSH and LH receptors (A5, A6), and reduces the expression of estrogen receptors (A7). Estrogen signalling enhances the production of aromatase (A8), as well as the production of the receptors for FSH (A9), LH (A10), and estradiol (A11). FSH receptor expression is observed also in the absence of FSH, LH, and estrogen signalling, and we therefore introduce a further regulation-independent component *ϑ*, which may represent testosterone-dependent signalling (A12). Blue arrows indicate exchange with the blood, black arrows indicate chemical reactions (binding or catalysis), green arrows indicate activating impacts and red arrows indicate inhibitory impacts. All components also decay, but for greater clarity decay reactions have not been included in the scheme. For a detailed description of the network interactions along with the evidence see the main text.

Ovarian follicular development has been studied for decades and has allowed major progress in animal breeding and assisted reproduction in the human. Many animal models of folliculogenesis are in use [1], partly in their own right to help the advancement of breeding and partly as models for human reproduction. Because of the bulk of published data dealing with bovine folliculogenesis and its great similarity to its human counterpart [2] we focused here on the bovine as a model system. As humans, horses and some sheep breeds, the cow is a mono-ovulatory species [3], i.e. one dominant follicle is selected from a cohort of 5-30 follicles [2,4]. All other recruited follicles undergo atresia [5]. The number of early growing follicles slowly declines with increasing age. Key regulators of follicular selection and maturation are the gonadotropins follicle-stimulating hormone (FSH) and luteinizing hormone (LH), both secreted in the pituitary [5]. While the initial, slow growth of follicles is independent of these gonadotropins, further progression to the antral follicular state requires FSH [6]. FSH induces the expression of a wide range of genes, including those of the LH-receptor and the aromatase, an enzyme that converts androgens into estrogens [7]. Within the follicle the regulatory interactions result in a complex network (Figure 1), with many of the components restricted spatially.

In spite of much detailed data, an integrated understanding of the processes regulating folliculogenesis is still lacking, and given the many incoherent feedbacks, several experimental findings appear counterintuitive. Theoretical models bear the potential to integrate large amounts of information into a consistent framework. A number of theoretical models have already been developed for ovarian follicle development (for a review see [8]), but only few models explore the processes within the follicle. Given the large size of both human and bovine follicles (15-20 mm), gradients are likely to form, and transport by diffusion may become limiting as indeed noted in models of oxygen transport in the follicle [9-11]. Since many of the signalling components are produced only in isolated parts of the follicle, with some diffusing and others being cell-bound, also spatio-temporal signalling gradients can be expected to emerge that may play an important role and may explain some of the counterintuitive data.

We therefore sought to build a reaction-diffusion model that would describe the signalling dynamics of the regulatory interactions between FSH, LH, estradiol, androgens, and insulin growth factors (IGF) in space and time. Quantitative data is available to determine virtually all parameter values, and the model is consistent with published data. The data-based model can be used to explore the patterning mechanisms inside the follicle, and to understand the molecular causes and effects of alterations observed in infertile patients.

## Methods

### Model development

Follicle development in the cow has been described in great detail [3,12] and much data is available to base the model on. We will focus on the development of the dominant follicle and leave the follicle selection process to future work. We aim at developing a parsimonious model for the process and thus seek to keep the regulatory interactions as simple as possible while reproducing the measurements. We focus on the hormones FSH (*F*), LH (*L*) and their receptors, on androgens (*A*) estradiol (*E*) and the estrogen receptor for steroid-dependent signalling, as well as on IGF signalling (*I*). The aromatase is not explicitly included in this parsimonious model because its activity can well be approximated as the direct result of IGF signalling and regulation through FSH and LH as discussed in detail below. Receptors of FSH, LH, and estradiol will be denoted *R*_*i*_ and the receptor complexes *C*_*i*_, with *i* = {*F*,*L*,*E*}. The modelled core network that regulates the development of the follicle is shown in Figure 1. Before we discuss the regulator interactions in detail we first introduce the geometry of the computational domain.

**Figure 2:**
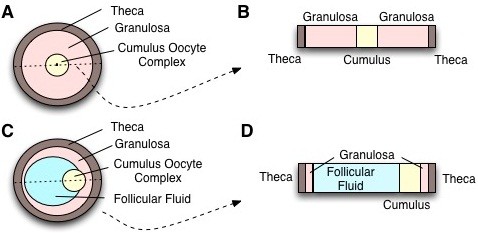
**The Geometry of the ovarian Follicle.** **(A)** In the follicle the oocyte is embedded in somatic tissue. The innermost layer are the cumulus cells (yellow) followed by a layer of granulosa cells (pink), and an outer layer, the theca (brown), that is enervated by blood vessels. Formation of androgens is restricted to the theca, while the formation of estradiol is restricted to the granulosa cells. **(B)** The computational domain represents a 1D cross-section of a follicle and expands over time. The outer part of the domain (brown) is the theca, the intermediate layers (pink) represent the granulosa; cumulus cells and the oocyte are in the center (yellow). **(C-D)** A more refined computational domain that also includes the follicular fluid (blue) on one side of the cumulus oocyte complex.

In contrast to human follicular development [13,14], the thickness of the granulosa layer of the bovine follicle has not yet been measured during its growth. Given the rotational symmetry, the fast mixing in the follicular fluid, and the lack of more detailed data, we restrict the model to a 1D cross-section through the follicle as illustrated in Figure 2A-D. Experimental measurements show that growth of the dominant follicle is about linear with time [15,16]. We can therefore approximate the growth process as a uniform expansion of the domain such that the length Λ(*t*) of the domain relates linearly to time, i.e.

(1) Λ(t)=Λ(0)+v×t,

where *v* is the growth speed of the domain. The domain is set to *x*(*t*) = Λ(*t*)*ξ* where *ξ* denotes the stationary coordinate frame *ξ* ∈ [−1, 1].

**Figure 3:**
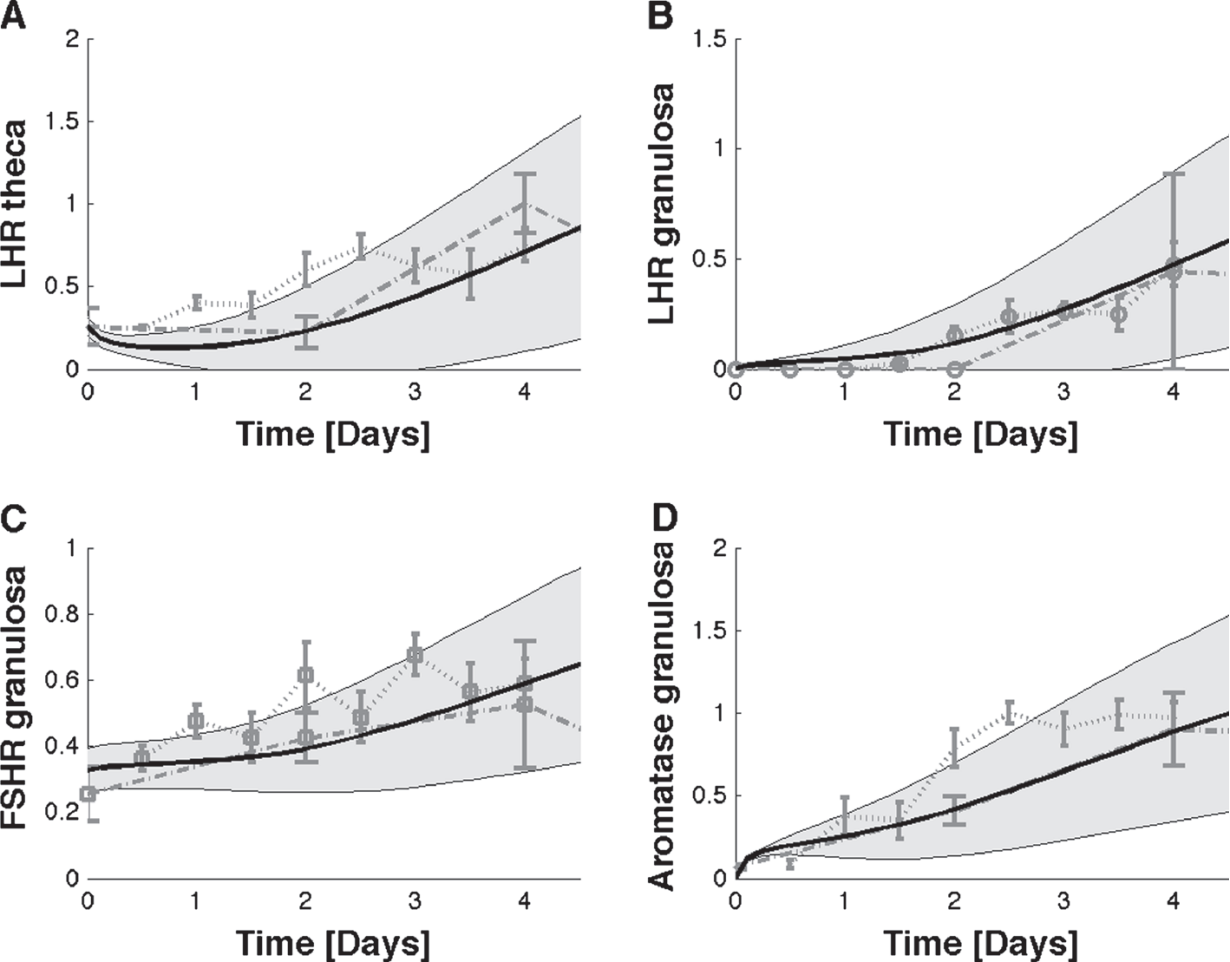
**Time-dependent expression profiles in the follicle.** Simulated and measured expression levels of LH receptor in granulosa and theca, and of the FSH receptor and the aromatase in the granulosa during the 1st wave of the bovine follicle maturation process. The data was recorded by [15,16]. The lower curves that extend to 10 days are the measurements in [16]; the follicles in these measurements were growing more slowly than in the study by [15]. Note that the dominant follicle undergoes atresia from day 6. **(A)** Data (dotted lines) and simulation predictions (solid line) of LH receptor expression in the theca. **(B)** Data (dotted lines) and simulation predictions (solid line) of LH receptor expression in the granulosa. **(C)** Data (dotted lines) and simulation predictions (solid line) of FSH receptor expression in the granulosa. **(D)** Data (dotted lines) and simulation predictions (solid line) of aromatase expression in the granulosa. The shaded area indicates the standard deviation of the simulations when noise is applied to the parameter values.

**Figure 4:**
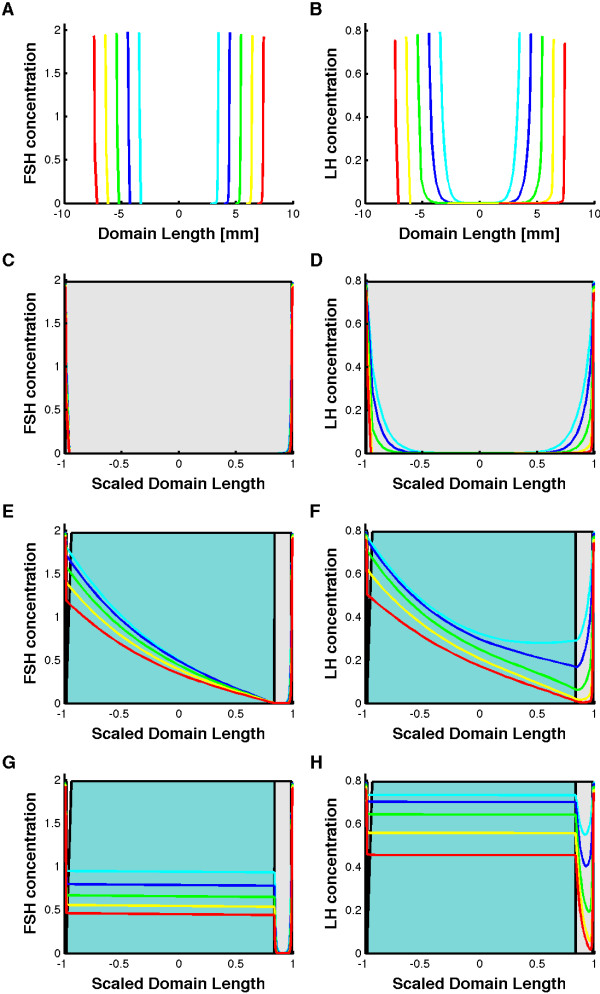
**FSH and LH gradient formation in the follicle.** At time zero no hormones are present in the follicle (black line). Over the next five days LH and FSH diffuse into the follicular domain from the boundary (theca) and form a gradient. The five time points are equally spaced at 0 (black), 1 (cyan), 2 (blue), 3 (green), 4 (yellow), and 5 days (red) curves. Panels **A** and **B** show the profiles on the growing domain. Panels **C** and **D** show the concentration profiles on a scaled domain. Panels **E** and **F** show the concentration profiles on a scaled domain if we include the fluid-filled antrum on one site of the COC block. Panels **G** and **H** show the concentration profiles on a scaled domain if we assume rapid mixing in the fluid-filled antrum. The shading indicates the different parts of the follicle, i.e. theca (white), granulosa cells (dark grey), cumulus cells (light grey), and follicular fluid (blue). Note that the theca and granulosa layers are very thin and thus barely visible.

**Figure 5:**
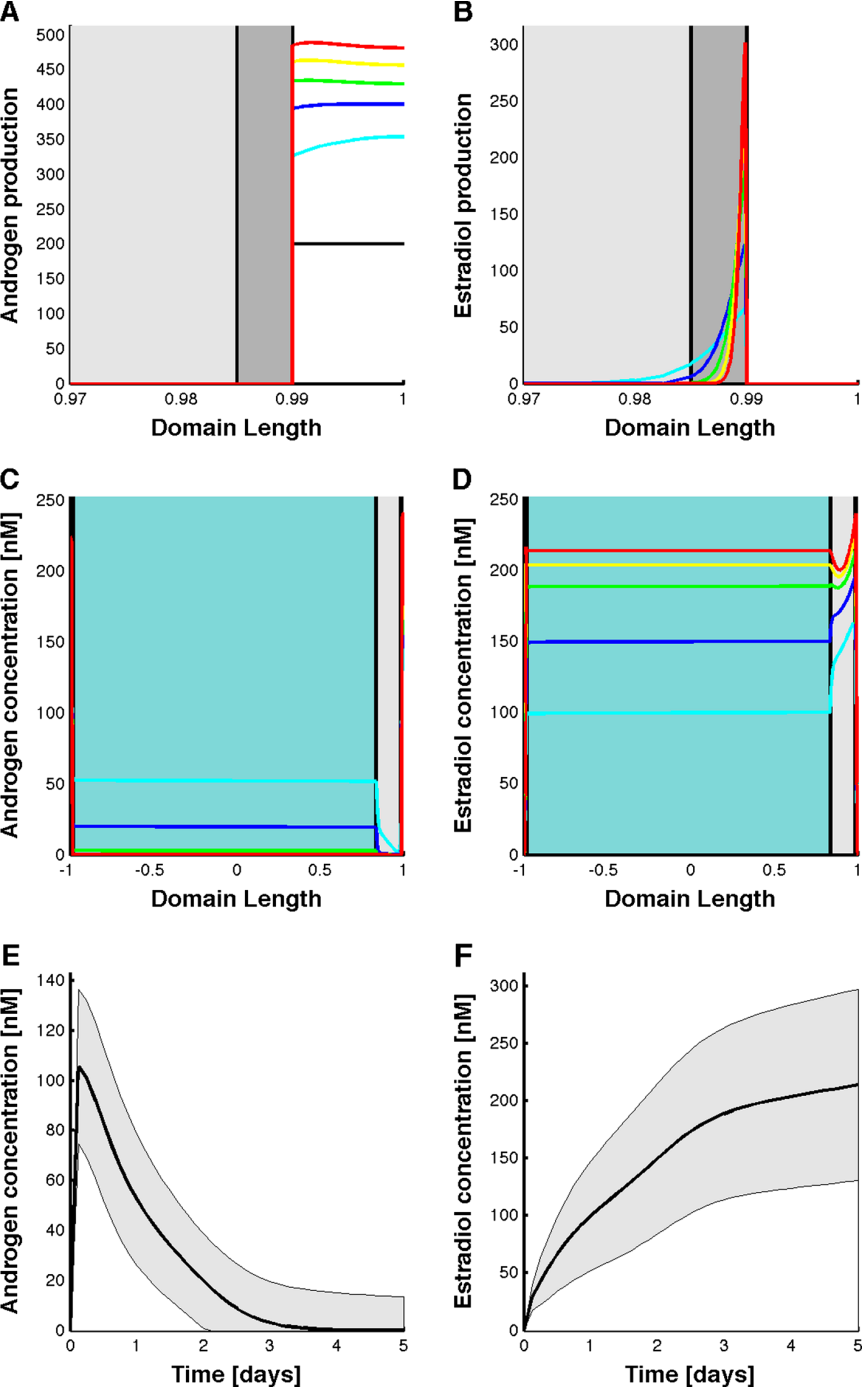
**The production of androgens and estradiol in the follicle.** **(A-B)** Production of (A) androgens and (B) estradiol. Only part of the domain is shown, i.e. theca (white), granulosa cells (dark grey), and a part of the COC (light grey). **(C-D)** Concentration profiles of (C) androgens and (D) estradiol. The five time points are equally spaced at 0 (black), 1 (cyan), 2 (blue), 3 (green), 4 (yellow), and 5 days (red) curves. All panels show the concentration profiles on a scaled domain. The shading indicates the different parts of the follicle, i.e. theca (white), granulosa cells (dark grey), COC (light grey), and follicular fluid (blue). Androgens are produced only in the theca, and estradiol is produced only in the granulosa cells. In the follicular fluid steroids are neither produced nor degraded. **(E-F)** The average steroid concentrations of (E) androgens and (F) estradiol in the follicular fluid over time. The shaded area indicates the standard deviation of the simulations when noise is applied to the parameter values.

**Figure 6:**
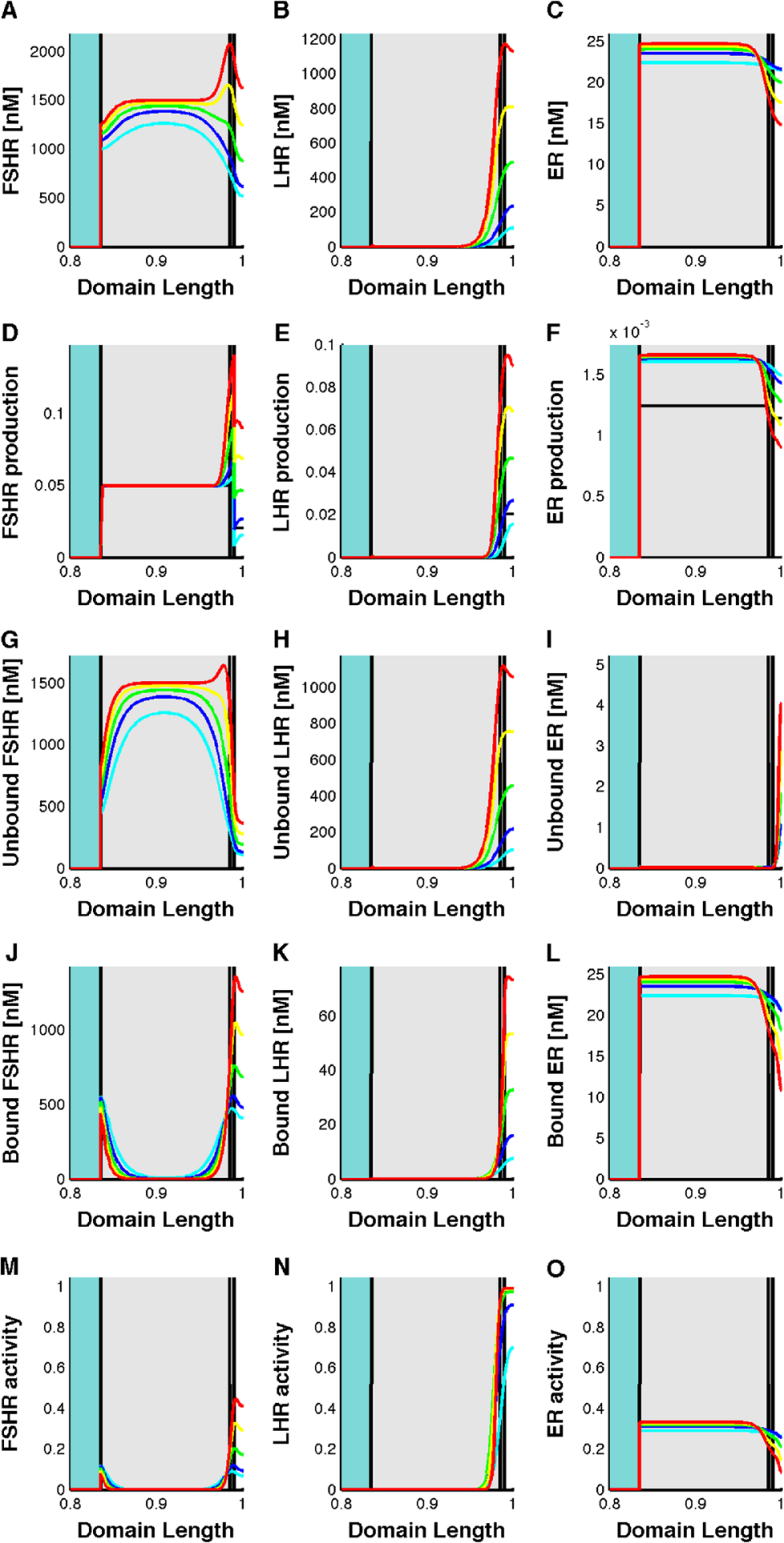
**The emergence of FSH-, LH-, and estrogen receptor gradients in the follicle.** **(A-C)** Receptor profiles of **(A)** FSH-receptor, **(B)** LH-receptor, **(C)** estrogen receptor. At time zero the receptor concentrations are low and the receptors are distributed homogenously in the follicle (black line). Over the next five days localised feedbacks create a graded distribution of the receptors. **(D-F)** Receptor production. **(G-I)** Unbound receptors **(J-L)** Bound receptors **(M-O)** Receptor activity. The five time points are equally spaced at 0 (black), 1 (cyan), 2 (blue), 3 (green), 4 (yellow), and 5 days (red) curves. All panels show the concentration profiles on a scaled domain. For better readability we only show the subset of the domain that includes the theca (white area), granulosa (dark shade), COC (light shade), and part of the follicular fluid (blue shade) on the right hand side of the domain. The shaded area indicates the standard deviation of the simulations when noise is applied to the parameter values.

At the beginning of follicular development the oocyte resides in the center of the follicle, surrounded by granulosa and theca cells (Figure 2A). At later stages a fluid-filled antrum emerges and the oocyte is thought to become located more at the periphery of the antrum of the follicle (Figure 2C). We will simulate both situations and discuss the impact of the fluid-filled cavity and the biased localisation of the oocyte. Certain reactions differ in the compartments. We therefore need to define compartment boundaries in the simulation. The thicknesses of theca and granulosa have been reported. In the first set of simulations we will only consider granulosa and theca (Figure 2A). Accordingly, we only need to define the position of the compartment boundary between granulosa and theca. The antral bovine follicle expands from a diameter of about 5 mm to about 20 mm [15,16]. The thickness of the theca interna measures only about 75 *μ*m in the dominant follicle, and the thickness of the theca remains constant [17]. The exact thickness of the theca is not relevant to the model because the blood flow determines the concentration of the soluble factors in this compartment and their distribution is therefore not diffusion-limited; the measured serum concentrations were reproduced by adjustment of the relative gain and loss rates. We can then make the simplifying assumption that the thecal layer scales over time, such that the border between the granulosa and the thecal layer stays at a constant position on the stationary domain, *ξ*_*θ*_ = 1 − 2 × 0.1/20 = 0.99. To show that this assumption is valid we include the plots, which are shown in Figures 3, 4, 5 and 6, with a 20% thicker thecal layer in the Supplementary Material (Additional file 1: Figure S 1, Additional file 2: Figure S 2, Additional file 3: Figure S 3 and Additional file 4: Figure S 4), using the same parameter values as before (Table 1). As can be seen, only the concentration of androgens, which are produced in the theca, increases significantly as the thickness of the thecal layer is increased (Additional file 3: Figure S 3); the overall gradient shapes remain very similar (Additional file 1: Figure S 1, Additional file 2: Figure S 2, Additional file 3: Figure S 3 and Additional file 4: Figure S 4). The small differences could be removed by adjusting the thecal production rates accordingly. The spatial restriction of reactions to theca (Θ) or granulosa (Γ) (as shown in Figure 2A,B) can then be incorporated by the use of a Heaviside function *H*, i.e.

(2) $$\begin{array}{l} \Theta =H(\xi^{2}-\xi_{\theta }^{2})\\ \Gamma =1-\Theta \end{array}$$

**Table 1:** Parameter values

	**Parameter**	**Simulation value**	**Reference value**	**References**
Parameter values directory from the literature	Λ(0)	5 mm	4-5 mm	[20]
	*v*	2.3 × 10^−5^ mm s^−1^	∼10 mm in 5 days	[20]
	*D*_*H*_	6.7 × 10^−5^ mm^2^ s^−1^	Average 6.7 × 10^−5^ mm^2^ s^−1^ for FSH	[21,22]
	*D*_*S*_	10^−4^ mm^2^ s^−1^	Small molecule Diff coeff 10^−4^ mm^2^ s^−1^	[23,24]
	$D_{C_{L}}$	2 × 10^−8^ mm^2^ s^−1^	1.9 ± 1 × 10^−8^ mm^2^ s^−1^	[25]
	*D*_*R*_	10^−7^ mm^2^ s^−1^	10^−9^−5×10^−7^ mm^2^ s^−1^	[26-29]
	*D*_*I*_	4 × 10^−9^ mm^2^ s^−1^	IGF receptor complex almost immobile	[26]
	*k*_*on*_	10^−3^ nM^−1^*s*^−1^	Standard value	[30]
	$k_{\text{off}}^{F}$	5 × 10^−4^ s^−1^	Average: *K*_*D*_ = 5 × 10^−10^*M*^−1^	[31-36]
	$k_{\text{off}}^{L}$	10^−2^ s^−1^	*K*_*D*_ = 9 nM	[37]
	$k_{\text{off}}^{E}$	10^−4^ s^−1^	*K*_*D*_ = 0.1 nM	[38]
	*δ*_*F*_	10^−5^ s^−1^	Half-life of FSH in ovariectomized ewes is 20 h	[39]
	*δ*_*L*_	5 × 10^−4^ s^−1^	Half-life of LH in ovariectomized rats is 23 minutes	[40]
	$\delta_{R_{F}}=\delta_{R_{L}}$	3 × 10^−5^ s^−1^	Measured decay rate 3 × 10^−5^ s^−1^	[41]
	$\delta_{C_{L}}$	7.5 × 10^−4^ s^−1^	Half-life LH/hCG-bound receptor 17 min	[40-42],
	$\delta_{C_{F}}=\delta_{C_{E}}=\delta_{E}=\delta_{A}$	6.4 × 10^−5^ s^−1^	Half-life is about 3 hours	[39,43-46]
	*δ*_*I*_	1.2 × 10^−6^ s^−1^	Half-life ∼ 10 hours	[47-49]
	$\rho_{R_{F}}=\rho_{R_{L}}$	500 × 0.5 pM s^−1^	21 receptors/cell/min = 500 × 0.5 pM s^−1^	[7,41,50-52]
	*ρ*_*E*_	0.06 s^−1^	*k*_*cat*_ = 0.06 s^−1^	[53,54]
	*K*_*M*_	44 nM	*K*_*M*_ = 44 nM	[53,54]
	*ρ*_*F*_	2 nM	FSH concentration ∼ 2 nM, flux Δ = 1 s^−1^	[55]
	*ρ*_*L*_	0.4 *ρ*_*F*_	LH concentration 40 *%* of that of FSH	[56]
Adapted	$\rho_{R_{E}}$	1.25 pM s^−1^	45000 estrogen receptors are detected per cell	[57]
	*ρ*_*I*_	2.8 *K*_*I*_*δ*_*I*_	Reproduce measured granulosa LH receptor numbers (≤ 3 nM)	[7]
	*ρ*_*A*_	100 nM	Reproduce androgen concentration in follicular fluid	[55,58]
	*K*_*I*_	5 *μ*M	Reproduce estradiol concentration in follicular fluid	[55,58]
Figure 3	*K*_*L*_	500 × 10 pM	Reproduce steepness of expression kinetics in Figure 3A	Figure 3A
	*K*_*F*_	500 × 3 nM	Reproduce steepness of expression kinetics in Figure 3B	Figure 3B
	*K*_*E*_	35 nM	Reproduce steepness of expression kinetics in Figure 3B	Figure 3B
	*ϑ*	0.2	Reproduce ordinate intercept in Figure 3C	Figure 3C
	*I*(0)	0.3*K*_*I*_Θ	Reproduce ordinate intercept in Figure 3A	Figure 3A

The table summarizes all parameter values used in the model along with the evidence. For details see the Parameters Section. In addition to the listed parameters we used *F*(0) = *L*(0) = *E*(0) = *A*(0) = *R*_*i*_(0) = *C*_*i*_(0) = 0 as initial conditions, Δ = 1 s^−1^ as exchange rate with the blood, and *n* = 2 as Hill coefficient. It should be noted that the factor 500 for the FSH and LH receptor production rates and Hill constants reflects the smaller membrane compartment (∼ 2.5 *μ*m^3^) compared to the entire cell (1140 *μ*m^3^[59]).

The quadratic term was included to represent the theca on both the positive and the negative x-axis (*x* ∈ Λ(*t*) × [−1, 1]).

When we include the follicular fluid in the simulations, the left part of the granulosa domain is reduced and follicular fluid is taking the space (Figure 2C). The antral bovine follicle expands from a diameter of about 5 mm to about 20 mm [15,16]. The thickness of the theca interna measures about 75 *μ*m [17], and the thickness of the parietal granulosa is about 50-65 *μ*m in dominant bovine follicles [17,18]. The cumulus oocyte complex (COC) in the mature, dominant human follicle measures about 3 mm in diameter. The remaining part is filled by the follicular fluid. We will not separately consider the granulosa cells that surround the oocyte as part of the COC, because, unlike in published research data dealing with human follicle development [19], such a distinction is not made in published research, on which the bovine model is based (i.e. [15,16]). The spatial restriction of reactions to theca (Θ), granulosa (Γ) or follicular fluid (Φ) (as shown in Figure 2C,D) can then be incorporated by a combination of Heaviside functions as

(3) $$\begin{array}{l} \Theta =H(\xi^{2}-\xi_{\theta }^{2})\\ \Gamma =H(\xi -\xi_{\phi_{1}})-H(\xi -\xi_{\phi_{2}})\\ \Phi =1-\Theta -\Gamma . \end{array}$$

As before the theca domain is set to *ξ*_*θ*_ = 0.99. The granulosa layer adjacent to the theca is also very thin compared to the diameter of the follicle (which measures about 20 mm in diameter), and measures about 50-65 *μ*m in bovine follicles [17,18]. Experiments reveal a broad distribution of follicular wall thickness in early follicles [18]. As the follicles grow with time, the distribution of follicular wall thickness appears to become more constant in the dominant follicles [17,18], whereas the thickness of the regressing follicles decreases [17]. In the absence of more detailed data on the relative expansion of the bovine granulosa layer we will assume that this layer scales with the expanding follicle, i.e. $\xi_{\phi_{1}}=-\xi_{\theta }+0.1/20=-0.985$. This implies a granulosa thickness of 12.5 *μ*m in the small 5mm follicles, which is at the lower limit of the observed distribution of granulosa thickness during early follicular growth. The COC in pre-ovulatory human follicles, which measure about 20 mm in diameter, is about 3 mm in diameter and is located adjacent to the mural granulosa. The follicular fluid fills the rest of the domain such that $\xi_{\phi_{2}}=-\xi_{\phi_{1}}-3/20=0.835$. All soluble proteins (but not the receptors) can diffuse into the follicular fluid. Given the absence of receptors in the follicular fluid, receptor-dependent degradation does not occur within the follicular fluid. Moreover, the, in comparison to the half-life of FSH, much shorter half-life of LH is attributed to unspecific cellular degradation processes that are triggered by the particular glycosylation of LH. Since cells are absent from the follicular fluid, we assume that LH is not degraded in the follicular fluid. Similarly, we assume that FSH, androgens, and estradiol are not degraded in the follicular fluid. This assumption is not critical for model conclusions because the concentrations in the follicular fluid can be adjusted by altering the production rates.

Since the hormones and their receptors can all diffuse within the follicle, if at different velocities, we formulate the model as isotropic advection-reaction-dispersion equations for a compound *c*_*i*_ with diffusion coefficient *D*_*i*_ and reaction terms $R(c_{i})$:

(4) $$\partial_{t}c_{i}+\nabla \left(uc_{i}\right)=D_{i}\nabla^{2}c_{i}+R\left(c_{i}\right)$$

where ***u*** denotes the external velocity field. Since we assume uniform growth on a 1D spatial domain we can solve a reformulated set of equations on a static domain *ξ* = [−1, 1] with *x* = Λ(*t*)*ξ*:

(5) $$\begin{array}{c} \frac{\partial c_{i}}{\partial t}+\frac{v}{\Lambda (t)}c_{i}=\frac{D_{i}}{\Lambda {\left(t\right)}^{2}}\frac{\partial^{2}c_{i}}{\partial \xi^{2}}+R\left(c_{i}\right). \end{array}$$

The reaction terms $R(c_{i})$ of the components describe the regulatory interactions based on information from the literature. We consider four classes of reactions: production, decay, complex formation, and catalytic processing as is discussed in detail below along with an introduction of all parameter names. The final set of equations for the reaction terms $R(c_{i})$ are:

(6) $$\begin{array}{c} R(F)=(\underset{\text{delivery}}{\underset{︸}{\rho_{F}}}\underset{\text{removal}}{\underset{︸}{-F}})\Delta \Theta \underset{\text{complex formation}}{\underset{︸}{-k_{\text{on}}FR_{f}+k_{\text{off}}^{F}C_{f}}}\underset{\text{decay}}{\underset{︸}{-\delta_{F}F}} \end{array}$$

(7) $$\begin{array}{c} R(L)=(\underset{\text{delivery}}{\underset{︸}{\rho_{L}}}\underset{\text{removal}}{\underset{︸}{-L}})\Delta \Theta \underset{\text{complex formation}}{\underset{︸}{-k_{\text{on}}LR_{l}+k_{\text{off}}^{L}C_{l}}}\underset{\text{decay}}{\underset{︸}{-\delta_{L}L}} \end{array}$$

(8) $$\begin{array}{c} R(A)=(\underset{\text{production}}{\underset{︸}{\rho_{A}(1+\sigma_{G})}}\underset{\text{removal}}{\underset{︸}{-A}})\Delta \Theta \underset{\text{decay}}{\underset{︸}{-\delta_{A}A}}\underset{\text{catalysis into estradiol}}{\underset{︸}{-\rho_{E}\Gamma \frac{A}{A+K_{M}}I(1+\sigma_{E}\sigma_{G})(1+\sigma_{G})}} \end{array}$$

(9) $$\begin{array}{c} R(E)=\underset{\text{removal}}{\underset{︸}{-E}}\Delta \Theta \underset{\text{complex formation}}{\underset{︸}{-k_{\text{on}}ER_{e}+k_{\text{off}}^{E}C_{e}}}\underset{\text{decay}}{\underset{︸}{-\delta_{E}E}}\underset{\text{catalytic production}}{\underset{︸}{+\rho_{E}\Gamma \frac{A}{A+K_{M}}I(1+\sigma_{E}\sigma_{G})(1+\sigma_{G})}} \end{array}$$

(10) $$\begin{array}{c} R(R_{f})=\underset{\text{production}}{\underset{︸}{\rho_{R_{f}}(\Gamma \vartheta +\sigma_{I}(1+\sigma_{E}))}}\underset{\text{complex formation}}{\underset{︸}{-k_{\text{on}}FR_{f}+k_{\text{off}}^{F}C_{f}}}\underset{\text{decay}}{\underset{︸}{-\delta_{R}R_{f}}} \end{array}$$

(11) $$\begin{array}{c} R(R_{l})=\underset{\text{production}}{\underset{︸}{\rho_{R_{l}}\sigma_{I}(1+\sigma_{E})}}\underset{\text{complex formation}}{\underset{︸}{-k_{\text{on}}LR_{l}+k_{\text{off}}^{L}C_{l}}}-\underset{\text{decay}}{\underset{︸}{\delta_{R}R_{l}}} \end{array}$$

(12) $$\begin{array}{c} R(R_{e})=\underset{\text{production}}{\underset{︸}{\rho_{R_{e}}(1+\sigma_{E})(1-\sigma_{I1})}}\underset{\text{complex formation}}{\underset{︸}{-k_{\text{on}}ER_{e}+k_{\text{off}}^{E}C_{e}}}\underset{\text{decay}}{\underset{︸}{-\delta_{R}R_{e}}} \end{array}$$

(13) $$\begin{array}{c} R(C_{f})=\underset{\text{complex formation}}{\underset{︸}{k_{\text{on}}FR_{f}-k_{\text{off}}^{F}C_{f}}}\underset{\text{decay}}{\underset{︸}{-\delta_{\text{CF}}C_{f}}} \end{array}$$

(14) $$\begin{array}{c} R(C_{l})=\underset{\text{complex formation}}{\underset{︸}{k_{\text{on}}LR_{l}-k_{\text{off}}^{L}C_{l}}}\underset{\text{decay}}{\underset{︸}{-\delta_{\text{CL}}C_{l}}} \end{array}$$

(15) $$\begin{array}{c} R(C_{e})=\underset{\text{complex formation}}{\underset{︸}{k_{\text{on}}ER_{e}-k_{\text{off}}^{E}C_{e}}}\underset{\text{decay}}{\underset{︸}{-\delta_{\text{CE}}C_{e}}} \end{array}$$

(16) $$\begin{array}{c} R(I)=\underset{\text{production}}{\underset{︸}{\rho_{I}\sigma_{G}}}\underset{\text{decay}}{\underset{︸}{-\delta_{I}I}} \end{array}$$

We use zero flux boundary conditions for all hormones, receptors and their complexes, i.e.

(17) $$\begin{array}{c} \nabla c_{i}=0. \end{array}$$

As initial conditions we use zero for the hormones, receptors and hormone-receptor complexes, because we intend to study the mechanisms that result in the emergence of the characteristic gene expression patterns in the follicle, i.e.

(18) $$\begin{array}{l} \;F(0)=L(0)=E(0)=A(0)=0\\ R_{F}(0)=R_{L}(0)=R_{E}(0)=0\\ C_{F}(0)=C_{L}(0)=C_{E}(0)=0. \end{array}$$

The only exception is the initial concentration of the IGF-receptor complex. IGF-2 and the IGF type receptor are expressed in the theca already at the time of antrum formation [60], and the early presence of the IGF-receptor complex in the theca is important in the model to reproduce the experimentally observed early expression of LH receptors in the theca [15,16]. To reproduce the measured LH receptor production rate in the follicle at day 0 as reproduced in Figure 3A we require

(19) $$I(0)=0.3\;K_{I}\Theta .$$

where Θ indicates the restriction to the theca and *K*_*I*_ is the Hill constant for IGF-dependent regulatory processes.

#### Detailed derivation of the reaction terms

In the following we discuss how the reaction terms in Eqs. 6-16 were derived from the results reported in the literature.

##### Exchange with the blood

FSH, LH, and steroid precursors are all produced outside the ovary and reach the theca via the blood, while estradiol is produced within the ovary and diffuses out off the ovary into the blood circulation [12]. The exchange with the blood results in a concentration flux in the theca. We describe this by including a constant source term for FSH, LH, and the androgen precursor in the theca, as well as a linear loss term for all soluble components (i.e. FSH, LH, androgens and estradiol) also in the theca. The exchange with the blood occurs at a rate Δ. Accordingly, the FSH, LH, and androgen delivery rates, *ρ*_*F*_, *ρ*_*L*_, and *ρ*_*A*_ are multiplied by Δ Θ (Eq. 3), i.e we have *ρ*_*F*_Θ Δ, *ρ*_*L*_Θ Δ, and *ρ*_*A*_Θ Δ in Eqs. 6-8. The delivery from the blood is balanced by a removal rate Θ Δ*c*_*i*_, that applies to all soluble components, i.e. *c*_*i*_ = {*F*,*L*,*E*,*A*}.

##### Decay, internalization and recycling

In the absence of contrary data we use the simplest model for decay, linear decay at rate *δ*_*i*_*c*_*i*_, where *δ*_*i*_ refers to the linear decay rate constant for component *i*. Internalized FSH and LH receptors do not recycle to the cell surface [42]. Potential recycling of estrogen receptors can be absorbed in the estradiol production rate, in particular because estrogen receptor signalling enhances estrogen receptor expression. To keep the model simple we thus do not include an independent term for estrogen receptor recycling.

##### Binding reactions

FSH, LH, and Estradiol all bind their respective receptors at rate *k*_*on*_ and unbind with a specific rate $k_{\text{off}}^{i}$,where *i* = {*F*,*L*,*E*}.

##### Regulatory interactions

The regulatory network is complex with many incoherent feedbacks and indirect regulatory interactions. Thus, production of FSH, LH and estrogen receptor are all enhanced by FSH, LH, and estradiol signalling [61-64]. However, many of these effects will reflect the multiple feedbacks between these components rather than direct auto-activation. These regulatory interactions can be at least in part be entangled with the help of mutant phenotypes and cell culture experiments. Thus, if expression of a gene is still observed in a certain mutant, then it is clear that this factor is not strictly necessary for the expression of the gene, but just enhances its expression. In that case the rate is proportional to 1+ *f*(*c*_*i*_), where *f*(·) denotes the particular functional relationship of the regulation and the +1 term enables the independent regulation of gene expression by other factors. Likewise if expression of a gene is observed only with a long delay after exposure to a certain factor it is likely that this factor does not act directly, but first up-regulates other factors that drive expression of the gene of interest. Such delays are important and need to be incorporated by introducing the intermediary factor.

Some of the processes are modulated by other signalling components and we use Hill functions to describe such regulatory influences. To describe activating influences of a component *c*_*i*_ we write

(20) $$\sigma_{i}=\frac{c_{i}^{n_{i}}}{c_{i}^{n_{i}}+K_{i}^{n_{i}}}.$$

and we use 1 − *σ*_*i*_ to describe inhibitory impacts of *c*_*i*_. Here *c*_*i*_ denotes the concentration of component *i*. *K*_*i*_ is the Hill constant which specifies the concentration of *c*_*i*_ where half-maximal activity is observed, and the Hill coefficient *n*_*i*_ defines the steepness of the response.

In the following we will discuss the reported evidence and how this was translated into the production terms.

FSH AND LH SIGNALING: Both the LH- and FSH-receptors are rhodopsin-like G-protein coupled receptors with great sequence identity [65] and connect to the same signalling machinery [66-69]. It is therefore possible that the observed differences in signalling outcomes mainly reflect ligand availability rather than distinct signalling programs by the receptor. We follow and test this possibility by making all regulatory impacts that have been reported for either LH or FSH signalling depended on both, i.e. instead of writing either *σ*_*F*_ or *σ*_*L*_ we will always write *σ*_*G*_ = *σ*_*F*_ + *σ*_*L*_.

IGF SIGNALING: The regulation of IGF signalling is complex with several ligands, receptors, and modulating binding proteins and proteases being part of the regulatory network. In follicles only expression of IGF-2 but not that of IGF-1 is detected, and expression of IGF-2 is restricted to the theca [60,70]. The level of IGF-2 expression seems not to change much during follicular development [60]. Type 1 IGF receptor mRNA is detected in both granulosa and theca cells of preantral and antral follicles, but expression is stronger in granulosa compared to the theca [60]. The circulating IGF concentration in healthy adults is ∼100 nM and the IGF type I receptor on most cells is typically approaching saturation at a concentrations of 5 nM or lower [71]. It has therefore been suggested that IGF activity is mainly regulated through IGF binding proteins (IGFBP), which sequester IGF in inactive complexes rather than through differences in the secretion of IGF. The IGFBP4 mRNA is selectively expressed by LH receptor (LHR) mRNA positive theca interna cells of healthy antral follicles (defined by aromatase and gonadotropin receptor expression) and by LHR expressing granulosa cells, which are present in large preovulatory and ovulatory follicles only (as defined by size and aromatase expression) [70]. In the mouse and the rat FSH stimulates the expression of PAPP-A, a metalloprotease which degrades IGF-B4 [72]. However, no such an effect was observed in bovine follicles [73]. The PAPP-A mRNA is abundant in granulosa cells of most ovarian follicles without obvious relation to IGFBP4 expression [70]. IGFBP4 mRNA levels are markedly increased after treatment with the LH analog, human chorionic gonadotropin (hCG), whereas the expression levels of IGF-2 and PAPP-A are not significantly altered [70]. In summary, there is evidence only for IGFBP4 being (positively) regulated by LH, while all other components appear to be expressed at a constant level. Since IGF-2 is expressed only in the theca, IGF signalling in the granulosa must be the result of diffusion. The diffusion coefficient of IGF-1 in cartilage has been established as *D* = 2.6 × 10^−5^ mm^2^ s^−1^. The diffusion length is determined also by the rate of ligand removal, by either receptor binding or unspecific degradation; the half-life of circulating unbound IGF has been found to be less than 2 min [74]. The characteristic length of such a gradient would be very short, i.e $\lambda =\sqrt{\frac{D}{k}}\leq 70\;\mu$m. The IGF binding proteins protect IGF from receptor binding and can thereby help to shuttle IGF into the follicle. The half-life in the presence of IGF binding proteins is increased some 100-fold by IGF binding proteins to several hundreds minutes, and as a result the gradient will some 10-fold wider. Thus, even though IGF binding proteins sequester IGF and remove it from the available active pool they also enable its transport into the follicle. Instead of simulating all those aspects in detail, we simplify the regulatory interactions in that we follow only the concentration of the active IGF-receptor complex which we denote be *I*.

##### Production and catalytic conversions

IGF RECEPTOR COMPLEX PRODUCTION: The concentration of the active IGF complex is enhanced by FSH and LH signalling [60,72,75], and accordingly we write for the rate of its generation *ρ*_*I*_*σ*_*G*_ in Eq. 16. Estradiol has been shown to both enhance and decrease IGF-1 expression in different experimental systems [76,77] and its effects will therefore not be included here.

LH RECEPTOR PRODUCTION: FSH and LH signalling results in the expression of a protein that limits the half-life of their mRNAs [78-80], such that FSH and LH receptors can only transiently (i.e. for a few hours) up-regulate their expression [63,64]. Thus, LH-dependent induction of increased LH receptor density is present after 2 h, but vanishes after 6h [81]. We will ignore this brief spike of auto-regulation and assume that there is no direct auto-regulation of FSH and LH receptor on their own expression on the relevant time scale (i.e. days). FSH-dependent LH receptor expression is enhanced by estradiol signalling, and LH receptor expression is reduced (but not absent) in response to the FSH analog in estrogen receptor-null follicles [82]. The rate of LH receptor expression must thus be proportional to (1 + *σ*_*E*_). FSH-dependent LH receptor expression cannot be triggered by estradiol in the absence of FSH [16,63] and the expression rate must therefore directly be dependent on an FSH-dependent term. FSH-dependent LH receptor expression is delayed [7,15,16]. Thus, stimulation with FSH increased the LH receptor density 10-fold within two days, with little change observed after 1 day [7]. FSH thus appears to act indirectly, possibly via IGF (*I*) or prolactin signalling [81]. IGF signalling is included explicitly in the model and any possible effects of other signalling systems such as prolactin can be considered to be implicitly represented by this variable. The delayed impact of FSH-dependent signalling of LH receptor expression can then be captured by making the LH receptor expression rate proportional to *σ*_*I*_(1 + *σ*_*E*_). FSH-dependent IGF-1 and LH receptor production is inhibited in cumulus cells by factors secreted by the oocyte [83,84] and LH receptor expression has been reported to be virtually absent in cumulus cells [83]. However, more recent experiments that use more sensitive techniques revealed the expression of the LH receptor in cumulus cells [19,85]. We therefore permit LH receptor expression throughout the follicle. In summary, we have $\rho_{R_{l}}\sigma_{I}(1\;+\;\sigma_{E})$ for the LH receptor production rate in Eq. 11.

FSH RECEPTOR PRODUCTION: As discussed above, FSH and LH signalling results in the expression of a protein that limits the half-life of their mRNAs [78-80], such that FSH and LH receptors can only transiently (i.e. for a few hours) up-regulate their expression [63,64]. Also in case of FSH receptor we will ignore this brief spike of auto-regulation and assume that there is no direct auto-regulation of FSH and LH receptor on their own expression on the relevant time scale (i.e. days). Similar as for the LH receptor IGF signalling has been shown to prolong the half-lifes of the FSH receptor mRNAs [72,86]. The IGF effect is observed only after a delay, i.e. IGF has been described to enhance FSH-dependent FSH receptor expression on rat granulosa cells after about 3 days of exposure [72] as is consistent with its delayed induction in granulosa cells. Also much as in case of the LH receptor, estradiol enhances FSH receptor expression on rat granulosa cells [87]. Thus, similar as for the LH receptor, the rate of FSH receptor expression should be $\rho_{R_{f}}\sigma_{I}(1+\sigma_{E})$. Unlike for the LH receptor, FSH-dependent FSH receptor expression is strongly reduced in IGF-null granulosa cells, but not absent [88], and FSH receptor expression in response to FSH or LH analogs was not impaired in estrogen receptor-null follicles [82]. There must therefore also be a FSH/LH/estrogen-independent regulation of FSH receptor expression, possibly triggered by testosterone signalling which has been described to induce the expression of FSH receptor and of aromatase [89]. The testosterone receptor is restricted to granulosa cells [90] and given the high steroid concentrations within the follicle the ligand-receptor complex can be assumed to have a constant activity *ϑ*. Here we ignore any possible feedback signalling that may up-regulate the expression of the receptor. In summary, we write for the FSH receptor expression rate $\rho_{R_{f}}(\Gamma \vartheta +\sigma_{I}(1+\sigma_{E}))$ in Eq. 10.

ESTROGEN RECEPTOR PRODUCTION: In the mouse estrogen receptor beta is the main form expressed in the follicles and is found mainly in granulosa cells, but has also been detected in the theca [82,91]. In bovine and sheep follicles both estrogen receptor alpha and beta are expressed in the granulosa and to a lesser extent in the theca [92,93]. In humans mainly estrogen receptor beta is detected in the granulosa cells, but also low levels of estrogen receptor alpha expression has been detected [94]. Estrogen receptor beta expression is enhanced by estradiol signalling and the production rate should therefore be proportional to (1 + *σ*_*E*_) [61,95]. There are also reports that FSH signalling inhibits estrogen receptor beta expression [61,95]. Since estrogen receptor beta is expressed mainly in granulosa cells FSH signalling may act indirectly via IGF signalling which is prominent in the theca; IGF receptor signalling has been shown to reduce estrogen receptor beta expression [96]. Accordingly, the estrogen receptor expression rate needs to be extended to (1 + *σ*_*E*_)(1 − *σ*_*I*_) to include this inhibitory effect. While LH-dependent signalling appears to interfere with IGF activity by inducing the expression of IGFBP4 mRNA [70] this additional feedback will be ignored to keep the model as simple as possible. In summary, we have $\rho_{R_{e}}(1+\sigma_{E})(1-\sigma_{I1})$ for the estrogen receptor production rate in Eq. 12.

ANDROGEN PRODUCTION: LH receptor signalling enhances the production of androgens in the theca [15], but only 25-50% of androgens is produced in the ovary (varying through the menstrual cycle) [53]. We therefore write for the androgen production term *ρ*_*A*_Θ(1 + *σ*_*G*_) in Eq. 8. It should be noted that despite the inclusion of FSH signalling, FSH will impact very little on androgen production because of the very low abundance of FSH receptors in the theca. It should be noted that high concentrations of LH appear to inhibit androgen production [97], but we ignore this to keep the model as simple as possible while reproducing the data.

ESTRADIOL PRODUCTION: Aromatase converts androgens into estradiol [98]. No feedback activity involving aromatase has been reported. To keep the model as simple as possible we therefore do not explicitly include the aromatase in the model, but rather incorporate the regulatory links that impact on its activity. Both the expression and the activity of aromatase are enhanced by FSH and LH signalling in the granulosa cells [7,98,99]. Estrogen receptor signalling enhances FSH-dependent expression of aromatase [82], and IGF signalling is necessary for FSH-dependent expression of aromatase [88,100] and enhances FSH-dependent estradiol production [101]. Testosterone signalling has been described to induce the expression of FSH receptor and of aromatase [89]. There is thus also some FSH/IGF-independent expression of aromatase. However, since based on data (Figure 3D) the effect is small we ignore this contribution and take the aromatase expression rate to be proportional to *I*(1 + *σ*_*E*_*σ*_*G*_). The activity of the aromatase is enhanced by FSH and should therefore be proportional to *I*(1 + *σ*_*E*_*σ*_*G*_)(1 + *σ*_*G*_). Accordingly, we then have according to the Michaelis-Menten law for the estradiol production rate in Eq. 9, $\rho_{E}\Gamma \frac{A}{A+K_{M}}I(1+\sigma_{E}\sigma_{G})(1+\sigma_{G})$, where *K*_*M*_ is the Michaelis-Menten constant; Γ indicates the restriction of the aromatase to granulosa and cumulus cells [102]. FSH and LH-dependent responses have been shown to be concentration-dependent: low concentrations of FSH and LH act stimulatory while high concentrations have negative impact on steroid production [97]. To keep the model as simple as possible while reproducing the data we will not include this feedback here; it would likely increase robustness to parameter value changes.

### Parameters

In spite of the large number of parameters, the model is rather tightly constrained because an enormous amount of data from cows and other animals is available that determine the parameter values. Where possible we use the measurements from cows. Table 1 summarizes all parameter values used in the model along with the evidence. The first 28 parameter values have been directly measured in experiments, 4 further values can be directly inferred from information in the literature, and the remaining 4 were set to fit the data in Figure 3, as was the initial condition *I*(0). In addition to the parameters listed above we used *F*(0) = *L*(0) = *E*(0) = *A*(0) = *R*_*i*_(0) = *C*_*i*_(0) = 0 as initial conditions to study the emergence of pattern from zero initial conditions. Furthermore, the exchange with the blood is modelled by a constant exchange rate, Δ. The exchange rate with the blood is not known, but as long as Δ ≥ 1 s^−1^ the flux does not impact on the predicted expression patterns in the follicle; we use Δ = 1 s^−1^. Finally the Hill coefficient in the regulatory terms was set to *n* = 2 throughout as in our previous models of developmental signalling networks [103-105]. In the following we discuss the literature on the parameter values in detail.

#### Length and time scales

In both humans and cattle 2-3 waves of follicle maturation occur until the dominant follicle survives and becomes ovulated [20,106]. We simulate the first wave of bovine folliculogenesis and focus on the FSH-dependent developmental phase of the dominant follicle, which lasts about 4-6 days before the dominant follicle of the first wave undergoes atresia [20]. The final day of the first wave is already characterized by atresia of the dominant follicle [16], and accordingly we include only the gene expression data of the first four days in Figure 3. The follicle is initially 5mm in diameter and expands over 4-6 days to 15-20 mm [20]. Based on the experimental observation that the follicle grows by about 10 mm in 5 days we use *L*(0) = 5mm and *v* = 2.3 × 10^−5^ mm s^−1^.

#### Diffusion coefficients

The diffusion constant of FSH has been determined as 6 × 10^−5^ mm^2^ s^−1^ for sheep FSH [21] and 7.43 × 10^−5^ mm^2^ s^−1^ for porcine FSH [22]. Accordingly, we will use the average of 6.7 × 10^−5^ mm^2^ s^−1^ as FSH diffusion coefficient. Given the great similarity of FSH and LH the same diffusion coefficient will also be used for LH. Steroids are very small and their diffusion coefficient is therefore set to *D*_*S*_ = 10^−4^ mm^2^ s^−1^, a typical value for small, diffusable compounds in aqueous solutions [23,24]. The diffusion coefficient of the LH-bound receptor complex has been established as 1.9 ± 1.0 × 10^−8^ mm^2^ s^−1^[25]. Interestingly, the diffusion coefficient of hCG-bound receptor complex was less than 10^−9^ mm^2^ s^−1^. Deglycosylated hCG-bound receptor complexes had a similar diffusion coefficient as LH-bound receptor complexes and were internalized also at the 50-times faster rate that is characteristic for LH-bound receptors. Accordingly. we have $D_{C_{L}}=2\times 10^{-8}$ mm^2^ s^−1^, which is more than 1000-fold lower than the diffusion coefficient for the ligand. The diffusion coefficient of the insulin receptors has been established as (3 − 5) × 10^−8^ mm^2^ s^−1^ at 23 degree Celsius [26]. Increasing the temperature to 37 degrees Celsius resulted in rapid receptor immobilization; the immobilization was attributed to aggregation of hormone-receptor complexes, their internalization, or a combination of both processes [26]. For simplicity we did not consider the unbound and bound IGF receptors separately in the model. To account for this we do not set the diffusion coefficient for the IGF receptor complex to zero, but to a 10-fold lower value than measured at 23 degree Celsius, i.e. *D*_*I*_ = 4 × 10^−9^ mm^2^ s^−1^. The diffusion coefficient of the FSH receptor has been measured as 4.4 × 10^−5^ mm^2^ s^−1^[107], which is unusually similar to that of the soluble protein, but at the upper end of what has been measured for membrane receptors [27-29]; the typical diffusion coefficient for unhindered diffusion in the membrane is about 10^−7^ mm^2^ s^−1^[27-29,108]. Since diffusion of the FSH receptors, much as diffusion of the cytoplasmic estrogen receptors will be restricted by the cell boundaries, even if cellular diffusion was very fast, we will use *D*_*R*_ = 10^−7^ mm^2^ s^−1^, the diffusion coefficient for unhindered membrane diffusion [27-29,108], for all other receptors. The use of a continuum description is reasonable in spite of the spatially restricted diffusion due to cell boundaries the diffusion coefficient is sufficiently low such that the cell-restricted proteins diffuse less than a cell diameter (∼7−10 *μ*m) within their half-life as discussed in detail before [104].

#### Binding rates

To avoid numerical problems we formulated our model for nanomolar concentrations rather than the SI standard of molar concentrations. The on-rates *k*_*on*_ are then 10^−3^ nM^−1^*s*^−1^[30]. The affinity constants for the ligand-receptor interactions have been independently determined in a range of different species and tissues and the reported values are very similar. The affinity constant for the FSH-receptor interaction has been determined as *K*_*a*_ = 1.5 ± 0.3 × 10^9^*M*^−1^ (*K*_*d*_ = 6.7 × 10^−10^*M*^−1^) [31] and *K*_*d*_ = 9.8 × 10^−11^*M*^−1^[32] in bull testes, as *K*_*d*_ = 6.7 × 10^−10^*M*^−1^ in rat testes [33], and *K*_*d*_ = 9.4 × 10^−10^*M*^−1^[34], *K*_*a*_ = 3 × 10^9^*M*^−1^ (*K*_*d*_ = 3.4 × 10^−10^*M*^−1^) [35], and *K*_*d*_ = 1.5 × 10^−10^*M*^−1^[36] for the human FSH receptor. The average measured *K*_*d*_ = 5 × 10^−10^*M*^−1^ corresponds to an off-rate $k_{\text{off}}^{F}=5\times 10^{-4}$ s^−1^. Human LH binds the rat LH receptor with *K*_*D*_ = 0.09 nM and the human LH receptor with a 100-fold lower affinity, *K*_*D*_ = 9 nM [37]. hCG binds the human LH receptor with a similar affinity, i.e. *K*_*D*_ = 4 nM [37]. Accordingly, we use $k_{\text{off}}^{L}=10^{-2}$ s^−1^. Estradiol binds its receptor with *K*_*D*_ ∼ 0.1 nM [38] which corresponds to $k_{\text{off}}^{E}=10^{-4}$ s^−1^. It should be noted that affinities and half-lifes have been reported to vary due to different glycosylations [56], but such details are beyond the scope of this study.

#### Decay and removal rates

The half-life of LH has been determined in ovary-intact rats as 13.7 ± 0.7min [40]. The half-life increases to 23.1 ± 2.9 min in ovariectomized rats [40]. While the degradation of LH in the ovariectomized rats will also reflect receptor-dependent degradation in other organs, 23 min must represent the lower limit for the receptor-independent half-life for LH (i.e. *δ*_*L*_ ≤ 5 × 10^−4^ s^−1^). The half-life of LH in ovary-intact rats of 14 minutes ( $\delta_{C_{L}}=8\times 10^{-4}$ s^−1^) is similar to the measured half-life of the hCG-stimulated human LH receptor of 17 min [42], and corresponds well to the decay rate of LH-bound LHR in MA-10 strains, a clonal strain of mouse Leydig tumour cells, which is 7 × 10^−4^ s^−1^[41]. In summary we will use the average rate $\delta_{C_{L}}=7.5\times 10^{-4}$ s^−1^ for the receptor-dependent rate of LH clearance. The turnover rate for unbound LH receptor has been determined as $\delta_{R_{L}}=3\times 10^{-5}$ s^−1^, and the intracellular degradation rate is 9 × 10^−5^ s^−1^[41].

The half-life of FSH is about 3h in ovary-intact ewes and about 20 hours in ovariectomized ewes [39]. While the degradation of FSH in the ovariectomized ewes will also reflect receptor-dependent degradation in other organs, 20 hours represents the lower limit for the receptor-independent half-life for FSH, and we use *δ*_*F*_ ∼ 10^−5^ s^−1^. The receptor-dependent half-life of 3h corresponds to *δ*_*Cf*_ = 6.4 × 10^−5^ s^−1^. Given the great similarity of the LH and the FSH receptor we use the same ligand-independent turnover rate, i.e. $\delta_{R_{F}}=\delta_{R_{L}}=3\times 10^{-5}$ s^−1^.

The half-life of androgens and estradiol has been reported as 2-3 h [43-45]. The half-life of the estrogen receptor-alpha protein has been reported as 3 h in the absence of estrogen, and as 1h upon addition of the hormone [46]. ERa half-life subsequently increases over time, achieving a half-life of ∼6 h in 72 h of estrogen treatment [46]. In summary we use, similar as for the FSH receptor complex, 3h as half-life for the steroid components, i.e. $\delta_{A}=\delta_{E}=\delta_{R_{E}}=\delta_{C_{E}}=\delta_{C_{F}}=6.4\times 10^{-5}{\text{s}}^{-1}$.

The half-life of overexpressed IGF receptor complexes has been measured to be much larger than 6 hours and smaller than 16 hours [47], which would correspond to a decay rate of about 2 × 10^−5^ s^−1^. In a subsequent study the same group reported a decay rate of 8 × 10^−5^ s^−1^ using cells that expressed 10^5^−10^6^ receptors per cell [48]. Granulosa cells have been reported to express 1125 ± 382 receptors per cell [109]. The rate of internalization was further reported to slow down substantially as insulin receptors become saturated, and half-maximal inhibition was observed already at 0.1 nM insulin concentrations; addition of 100 nM insulin reduced the internalization rate to less than 10% [48,49]. Based on these *in vitro* data the IGF receptor turnover rate in granulosa cells is difficult to estimate, but it is likely to be substantially lower than the measured 2 − 8 × 10^−5^ s^−1^, and we will use *δ*_*I*_ = 1.2 × 10^−6^ s^−1^.

Finally, the flux rate Δ determines the rate at which the soluble factors are delivered and removed from the follicle. This rate is not known. A low flux rate can lead to the removal and depletion of those soluble factors, which are delivered to the theca, i.e. FSH, LH, and the steroid precursor. A higher flux rate can in principle always be balanced by a higher production rate, but can cause numerical problems. To avoid such artefacts and problems we use as flux rate Δ = 1 s^−1^.

#### Production rates

The bovine serum FSH concentration during the follicular phase has been established as 66 ng/ml in one study [110] and as ∼20 ng/ml in several others [55,111-113]. If we use 1g ∼ 1 ml as done in similar studies before [114] and use 30 kDa as the molecular weight of FSH we arrive at a serum concentration of 0.7-2 nM for FSH. We note that the reported bovine FSH concentration is somewhat lower than the reported concentration in humans. Human FSH and LH concentrations are typically reported as IU/l which can be converted into molar concentrations based on reported conversions into protein weight per liter. Thus, dependent on the hormone standard used 1 IU corresponds to about 46 *μ*g FSH or about 23 *μ*g LH [115]. The molecular weight of both proteins is 30-35 kDa, depending on glycosylation. 1 IU/l FSH then translates into about 1.5 nM, and 1 IU/l LH translates into about 0.75 nM. FSH levels are high during the menstruation period (midcycle phase 4.5-22.5 IU/L) and lower in the middle of the cycle (follicular phase 3.9-8.8 IU/L and luteal phase 1.8-5.1 IU/L) [116,117]. In the human follicular phase the average FSH serum concentration has been reported as 5.3 IU/liter (8 nM) and the average LH concentration as 4.2 IU/liter (3.15 nM) [56]. In humans the serum LH concentration is thus about 40% that of FSH. The bovine LH concentration was determined as 0.63 ng/ml per liter (and thus 20 pM) [55]. The low reported LH concentration in the bovine study may reflect the short half-life of only 14 min [40] which makes accurate measurements of the LH concentration more difficult. In fact, in ewes the LH concentration has been reported as 25 ng/ml - 50 ng/ml LH [118] which would be in the expected range. The reported LH concentration in the bovine follicular fluid ranges from 0.02 to 0.63 nM [55,58] where the lower value was again reported by Rhind and co-workers. In conclusion, we suspect that the reported bovine LH serum concentration is underestimated. We therefore adjusted the production and loss rate in the theca such that the FSH concentration was 2 nM, the upper range of the reported bovine concentrations and the lower range of the reported human concentrations, i.e. *ρ*_*F*_ = Δ2 nM, and the LH concentration to 40% of this, i.e. *ρ*_*L*_ = 0.4*ρ*_*F*_. Oscillations in the dynamics of LH are ignored in this model. The impact of the LH/FSH concentration and ratio are explored in detail in the Results and discussion section.

The LH receptor production rate has been measured in a cell culture system as 21 receptors/cell/min [41] and the cell volume of human ovarian granulosa cells has been established as 1140 *μ*m^3^[59].This would then translate to $\rho_{R_{L}}=0.5$ pM s^−1^. The LH and FSH receptors are plasma membrane receptors and their reaction volume is therefore much smaller, even though both the oocyte and granulosa cells have elaborate numerous cytoplasmic projections and microvilli that interdigitate with each other to create an extremely large surface area for diffusion. Thus, the surface area of granulosa cells has been established by scanning electron microscope as 198.5 ± 6.3 *μ*m^2^ in unstimulated control rabbits and as 242.8 ± 9.28 *μ*m^2^ in rabbits injected 12 h earlier with hCG [119]. The effective binding volume for the receptors would thus be much smaller, i.e. 250 *μm*^2^ × 0.01 *μ*m = 2.5 *μ*m^3^ where we use a binding length of 10 nm as discussed elsewhere [120]. To take into account that the LH receptor is a membrane protein we thus need to use an about 500-fold higher receptor production rate, i.e. $\rho_{R_{L}}=250$ pM s^−1^ for the discrete receptor reaction volume. We note that while this number provides a better estimate for the effective binding kinetics, it greatly over-estimates the total amount of hormone that is removed, since we do not exclude the cell volumes (that are inaccessible to the hormones) from the simulations. Since the hormone concentration is kept constant in the theca this, however, does not affect any of our predictions. To include the effects of the membrane localisation of receptors a cell-based model would need to be devised.

The FSH receptor expression rate was set to that of the LH receptor, i.e. $\rho_{R_{F}}=\rho_{R_{L}}=250$ pM s^−1^. The activity of the FSH/LH-independent regulation of FSH receptor expression was set to *ϑ* = 0.2 to reproduce the ordinate intercept in Figure 3C.

The simulated LH receptor concentration also depends on the concentration of IGF receptor complexes and thus on *ρ*_*I*_. In cultured rat granulosa cells 200 LH receptor sites were detected per cell [7] which corresponds to a concentration of about 300 pM if we use 1140 *μ*m^3^ as granulosa cell volume [59] and 150 nM if we use the much smaller reaction volume of 2.5 *μ*m^3^ as discussed above. Stimulation with FSH increased the LH receptor density 10-fold within two days, with little change observed after 1 day [7]. The maximal LH receptor concentration would thus be 3 nM (or 1500 nM if we use the much smaller reaction volume) in our model. To achieve such a concentration range in the model we require *ρ*_*I*_ = 2.8 *K*_*I*_*δ*_*I*_.

We have found measurements of the estrogen receptor concentration only in the pituary of ewes. In cell culture systems about 45000 estrogen receptors are detected per cell [57]. This would correspond to about 65 nM and we therefore use $\rho_{R_{E}}=1.25$ pM s^−1^. It should be noted that the much larger estrogen receptor concentration reflects its cytoplasmic/nuclear rather than membrane localisation. This much higher concentration, however, does not impact the model predictions as long as the Hill constant *K*_*E*_ is adjusted accordingly.

Androgen production depends on the precursor concentration and on the stimulating impact of FSH and LH signalling. The most potent androgen, testosterone, is secreted by the adrenal zona fasciculata (25%) and the ovarian stroma (25%), with the remaining 50% being produced from circulating andostrenedione [53]. Androstenedione is secreted by the adrenal zona fasciculata (50%) and the ovarian stroma (50%, but varying through the menstrual cycle). The maximal follicular fluid concentrations of androstenoide and testosterone have been reported as 107 nM and 100 nM respectively [58]. Accordingly, we use as androgen production rate *ρ*_*A*_ = Δ × 100 nM, where 100 nM would be the concentration of the precursors.

Estradiol is produced from androgens by the cytochrome P450 enzyme aromatase. The human cytochrome P450-dependent conversion rate, the rate limiting step in estrogen production, has been established as *k*_*cat*_ = 0.06 s^−1^; *K*_*M*_ = 44 nM [54]. Accordingly we use *ρ*_*E*_ = 0.06 s^−1^ and *K*_*M*_ = 44 nM.

#### Hill constants and coefficients

The Hill coefficient *n* is not known, and much as in our previous studies of developmental signalling processes [104,105] we will use *n* = 2 throughout, which is in the likely physiological range [30] and which has the added benefit of rendering the simulation numerically more stable. Hill constants determine the concentration at which half the activity is attained. There are no direct measurements of the Hill constants, but these must lie within the dynamic range of the receptor-ligand complex concentration to enable the reported regulatory effect of the regulatory components. The Hill constant for the LH receptor determines the slope of the LH expression kinetics in Figure 3A and, to reproduce the data, needed to be set to *K*_*L*_ = 500 × 10 pM where the factor 500 reflects the smaller reaction volume of the membrane receptors as discussed in the previous section. This would correspond to about 10 active ligand-receptor complexes for half maximal activation. This number for the LH receptor is rather low. However, as discussed above the concentrations in the model are about 4-fold lower than in reality because we do not explicitly consider the restriction to the membrane. Moreover, receptors cluster on the membrane and thus increase their local concentration. The Hill constants of the FSH and estrogen receptors determine the slope of the LH expression kinetics in Figure 3B and needed to be set to *K*_*F*_ = 500 × 3 nM and *K*_*E*_ = 35 nM to reproduce the data. Also in case of the estrogen receptor the real cellular concentration will be higher because estrogen receptors move to the nucleus which has a smaller volume of about 250 *μ*m^3^[59]. There is thus a further concentration by compartmentalization. While the IGF receptor concentration is low (1.7 nM IGF receptors [109]), the IGF receptor complex is used to approximate the activity of the aromatase which likely has a much higher concentration, given its cytoplasmic/nuclear localisation. We require *K*_*I*_ = 5 *μ*M to obtain a final estradiol concentration of 200-250 nM in the follicular fluid. The value of *K*_*I*_ only affects the rate of estradiol production, but none of the other kinetics as long as *I*(0) = 0.3 *K*_*I*_, and *ρ*_*I*_ = 3*K*_*I*_*δ*_*I*_ are adapted accordingly.

### Numerical solution

The PDEs were solved with finite difference methods (pdepe) as implemented in MATLAB. The robustness of the model to small parameter variations was assessed by simultaneously adding Gaussian noise to all parameter values. To this end 100 simulations were run with parameter values drawn from a Gaussian distribution with mean values equal to those in Table 1 and with standard deviation 0.2 and the standard deviation in model output was calculated and included as shaded area in all figures where it does not reduce readability.

## Results and discussion

### Model consistency with data

In summary, the model consists of 11 variables and 36 independent parameters in addition to the initial conditions, zero flux boundary conditions, the flux term Δ, and the general Hill coefficient *n* (Table 1). In spite of the large number of parameters, the model is very much constrained by experimental data. Thus, for more than three quarters (28) of these 36 parameters the values were set according to reported measurements (Table 1). These represent the measured affinities of the ligand-receptor interactions, the protein half-lifes, production rates, as well as the growth rate of the follicle. All initial conditions except for that of the IGF signalling complex, I(0), were set to zero because we wanted the patterns to emerge from the regulatory interactions rather than being pre-set. I(0) was set as to reproduce the ordinate intercept in the LH expression kinetics in Figure 3A. The production rates that had not been directly measured as well as the response threshold of IGF signalling (Hill constant *K*_*I*_) were set as to match the reported concentrations. All other response thresholds (Hill constants) could be inferred from the measured gene expression time courses (Figure 3). We set the response thresholds for LH signalling (Hill constant *K*_*L*_) as to reproduce the slope in the time course of LH-receptor expression in the theca (Figure 3A) and the response thresholds for FSH and estrogen signalling (Hill constants *K*_*F*_, and *K*_*E*_) to reproduce the slope of the LH expression kinetics in the granulosa (Figure 3B). The activity of the FSH-/LH-independent expression of FSH receptors, *ϑ*, was set as to obtain the ordinate intercept in the measured FSH receptor expression kinetics (Figure 3C).

The delay in the emergence of the expression of the LH-receptor and of aromatase activity in the granulosa (Figure 3B,D) is the result of the slow emergence of IGF-receptor complexes in the granulosa. The time scale on which IGF-receptor complexes emerge is determined by its turnover rate *δ*_*I*_ = 1.2 × 10^−6^ s^−1^. For a shorter half-life the delay would be shorter, while for a longer half-life the delay would be even longer. While this rate has been determined by several research groups, the measurements have all been carried out with cell lines that overexpressed the IGF receptor at levels more than 100-fold higher than what has been observed in granulosa cells [47-49]. Moreover, the turnover rate was found to slow down as receptors become saturated by ligand [47-49]. As a result the experimentally determined rates of 2 − 8 × 10^−5^ s^−1^ presumably reflect a maximal possible turnover rate, rather than a physiological turnover rate. Given the importance of this rate for the signalling processes in the follicle this rate should be measured again in the natural environment.

In spite of these limitations in the determination of *δ*_*I*_, we note that the measurements, although obtained by many independent research groups, are largely consistent and also reproduce additional data very well. One example is provided by the slope in Figure 3C, which is determined by the rate of FSH receptor expression, *ρ*_*Rf*_, a rate that was set to be equal to the measured value for the LH-receptor [41]. Further aspects are also reproduced very well (Figures 4, 5 and 6) as discussed below.

While inaccuracies in the measurements are still possible, we note that the relative production and decay rates, the Hill constants, and the binding affinities are interdependent and inaccuracies in the directly measured parameter values are therefore largely compensated by the fitted parameters. As a result, we obtained similar results when we changed parameter values during the course of model development as long as we adjusted the 8 parameters discussed above such that the key characteristics were all still reproducable. Given the large number of parameters and studied outputs a global sensitivity analysis of such spatio-temporal model is impossible, in particular because, for the analysis to be informative, the 8 parameter values and the non-zero initial condition I(0) that were not measured directly would always have to be adjusted such that the model output would still match the measured concentrations (Table 1) and expression kinetics (Figure 3). In a model of lung development we have previously added Gaussian noise to all parameter values to study the effect of simultaneous variations in several parameter values [104], and with a similar approach we find that the overall model behavior is robust in the presence of moderate noise levels; shaded areas are included in all Figures where it does not reduce readability to indicate the standard deviation in the model output to Gaussian noise with standard deviation of 20%. Single parameter perturbations can be used to identify critical parameters, and such perturbations are particularly valuable when compared to similar experimental perturbations. No bovine transgenic data is available and murine folliculogenesis is very different [1].

### Hormone gradients in the follicle

Initially no hormones or receptors (except for IGF-receptor complexes) are present in our model. LH and FSH then diffuse into the domain from the boundary. As shown in Figure 4A,B we observe the formation of a gradient of both FSH and LH. If we plot the gradient on a domain where the size of the growing domain is scaled with respect to the current length of the domain L(t), we notice that the relative extension of the gradients shrinks as the domain expands (Figure 4C,D).

An important aspect, so far neglected, is the formation of the antrum, a fluid-filled cavity within the follicle. In our 1D-model we captured this by including a fluid-filled part of our domain lacking cells and as such receptor-free (Figure 2C,D). As a result diffusion is unhindered in that part of the domain (shaded blue) and the gradients extend further from the source in the theca to the sink in the granulosa and cumulus oophorus-oocyte complex (COC) (shaded grey) (Figure 4E,F). In fact, it is likely that the concentrations in the follicular fluid are homogenous since the aqueous fluid [121] will be mixed as the animal moves.

When we include such rapid mixing in the follicular fluid then the concentration gradients vanish in this compartment and the average concentrations in the follicular fluid increase (Figure 4G,H).

In both scenarios we predict a difference between the concentrations in the serum and in the follicular fluid (Figure 4E-H). The extent of this difference depends on how much hormone is bound by the receptors, and thus on the receptor concentration. The receptor concentration in turn depends on the size of the reaction volume, which we estimated to be some 500-fold smaller than the cell volume. For a smaller factor the concentration difference between follicular fluid and serum would be less pronounced. The bovine FSH concentrations have been reported to be similar in the serum (0.7-2 nM) and in the follicular fluid (0.6-2 nM), but the measured range is wide and the data were not acquired in the same animals [55,58,110]. In humans, such concentration differences between serum and follicular fluid has indeed been observed [122].

### Steroid production in the follicle

Androgens are produced from steroid precursors in the theca or reach the theca via the capillary blood vessels surrounding the follicle [53]. Estradiol is produced only in granulosa [7,98,123]. In the simulation androgen production was therefore restricted to the theca, while production of estradiol was restricted to granulosa (Figure 5A,B). The simulation further predicts that within the granulosa layer estradiol production is strongest close to the theca where the positive impact of IGF signalling on aromatase expression would be stronger and the androgen concentration higher (Figure 5B).

From the theca androgens either diffuse into the follicle or are removed from the follicle via the blood circulation. Accordingly, the androgen concentration is the highest in the theca and falls towards the centre of the follicle (Figure 5C). Similarly, the estradiol concentration is the highest in the granulosa and falls towards the follicular fluid and the theca (Figure 5D). Interestingly, within the follicular fluid the androgen concentration is predicted to decline (Figure 5E) while the estradiol concentrations are predicted to rise (Figure 5F) as the follicle becomes larger. Similarly, the androstenedione concentration was found to decline from 107 nM to 33 nM and the testosterone concentration was found to decline from 100 nM to 10 nM [58]. In the same experimental study the estradiol concentration in the follicular fluid was found to rise from 17 to 230 nM [58]. While the maximal testosterone concentration in the follicular fluid is determined by the precursor concentration and thus *ρ*_*A*_ and the maximal concentration of estradiol in the follicular fluid is determined by the activity of the aromatase and thus by *K*_*I*_, the decreasing testosterone and increasing estradiol concentrations themselves are not hard-coded and emerge from the regulatory interactions. It should be noted that while estradiol has been reported to down-regulate testosterone production [82] in the model the decline in the testosterone concentration in the follicular fluid is observed without such negative feedback on its production.

### A self-emerging spatial organisation of receptor distributions

The expression patterns of the FSH- and LH-receptor in bovine follicles over time have been reported [15,16]. FSH-receptors localize mainly to the granulosa [55] while LH-receptor are first present close to the theca and emerge later in the granulosa [15,16] with a declining gradient in LH-receptor expression towards the center of the follicle [83,124]. How this distribution emerges is not clear. The model now reveals that these observed receptor expression patterns result directly from the reported regulatory interactions. Thus, the model reproduces the high concentration of FSH-receptors in the granulosa cells (Figure 6A) and its much lower expression in the theca (Figure 5D). LH-receptors on the contrary initially mainly concentrate in the theca (Figure 6B,E, light blue line), later also in the granulosa and in the outer part of the COC (Figure 6B,E, green-red lines). The extent to which LH-receptors appear in the granulosa depends on the value of the Hill constant for the FSH-receptor, *K*_*F*_: the stronger the FSH-receptor signalling in the granulosa, the wider the LH-receptor distribution in the COC. Note that in Figure 6 we only show the subset of the domain that includes the theca, granulosa, COC and part of the follicular fluid.

Estrogen receptors are expressed in immature granulosa cells [95], but the expression of estrogen receptor-beta, the dominant form in the ovary, is mainly restricted to the granulosa cells of growing follicles [125,126]. Such a stronger expression in the granulosa emerges in our model as the result of the negative impact of FSH and LH signalling (via IGF signalling) on estrogen receptor expression. The stronger this feedback, the more restricted is the expression of the estrogen receptor to granulosa and COC (Figure 6C,F). We note that low expression levels of estrogen receptor in the theca are important to reproduce the physiological gene expression time course of LH-receptor expression. Some expression of the estrogen receptors in the theca has indeed been reported [127] and conditional knock-outs reveal an important role of estrogen receptor in the theca and absence of the receptor results in infertility in mice [126]. The mice are characterized by the presence of more antral follicles and failure to ovulate [126].

The FSH-receptor concentrations nicely demonstrate the consistency of the reported measurements. Even though the FSH-receptor expression rate *ρ*_*Rf*_ was set based on measured values and was thus not adapted to fit any particular feature of the model, the predicted FSH-receptor concentrations agree well with earlier measurements. Measurements in isolated granulosa cells reveal a density of 1500 FSH receptors by the secondary stage of follicular development and FSH-receptor numbers remains relatively constant during further development [50]. Available data in the human indicate that the number of FSH-receptors does not change during antral development, at least not until follicles reach a diameter of 12 mm [4]. Using a granulosa cell volume of 1140 *μ*m^3^[59] 1500 FSH-receptors correspond to 2.2 nM (and to 1100 nM if we used the small membrane reaction volume of 2.5 *μ*m^3^). This concentration is indeed observed with a 2-fold increase at later stages in the parietal granulosa (Figure 6A,D). Such delayed 2-fold increase in FSH-receptor expression is also observed in experiments [128]. The IGF production rate was adjusted to reproduce the measured number of LH receptors in isolated rat granulosa cells which would correspond to 0.3 nM if we use the entire cell volume or 150 nM for the smaller membrane compartment (Figure 6B) [7]. In the *in vitro* experiments the LH receptor density increased some 10-fold over 2 days in response to ligand stimulation [7]. A slow 10-fold increase in LH receptors also emerges from the regulatory interactions in the model (Figure 6E). The estrogen receptor expression rate was adjusted to obtain the typical estrogen receptor concentration of about 65 nM (Figure 6C,F) [57].

While estrogen receptors are saturated at the ligand concentrations available in the follicle (Figure 6I,L), ligand-bound LH-receptors are rapidly internalized, and the concentration of ligand-bound LH-receptors is therefore much lower than the total LH-receptor concentration (Figure 6H,K). About one quarter of all FSH-receptors remain unbound (Figure 6G,J). Data from the hamster indicate that approximately 1% of the FSH serum concentration is tissue-bound within the ovary [129], but these measurements are difficult to compare to the simulation results.

It is an open question as to how the signalling responses of FSH- and LH-receptors differ. The differential activity in the model is the consequence of the combined effects of different spatial gradients, different ligand-receptor affinities and their different Hill constants. The downstream signalling effects of the active receptors are equivalent. The activity of FSH signalling is mainly restricted to the granulosa cells but also extends to the theca (Figure 6M). LH signalling on the contrary first concentrates in the theca and then expands to the granulosa (Figure 6N). Estrogen receptor activity extends throughout the granulosa and COC, and is somewhat lower in the theca (Figure 6O).

## Conclusion

We have developed a 1D-computational model to integrate the large amount of published data dealing with bovine ovarian follicle development into a consistent spatio-temporal framework of ovarian folliculogenesis. A large amount of quantitative data is available that determined the parameter values in the constructed model (Table 1). As a result the model is highly constrained and reproduces biological observations, which were not explicitly included in the model and which were previously difficult to understand by simple verbal reasoning.

The spatio-temporal model reveals the importance of distances and gradients in the developing follicle. Because of the receptor-dependent removal of the hormones, their activity is strongly limited in fields of cells that express the receptor but not the ligand. Both human and bovine follicles grow to a similar final size of approximately 20 mm. If the follicles were filled entirely with receptor expressing cells and if the oocyte were located in the center of the follicle, then the cells close to the oocyte would not receive any endocrine signalling (Figure 4C,D). This limitation is overcome by the emergence of a fluid filled cavity and the localization of the COC in the periphery of the follicle (Figure 2C,D). As a result high concentrations of the exogenous hormones reach the COC (Figure 4E,F), in particular if we also take rapid mixing of the hormones in the fluid-filled cavity into account (Figure 4G,H).

In agreement with earlier experimental data the model predicts that estradiol is produced mainly in the parietal granulosa layer (close to the theca) (Figure 5A,B), because here the androgen concentration is higher and the stimulating impact of IGF-receptor complexes, and FSH- and LH-signalling stronger (Figure 6M-O). Moreover, in agreement with earlier experimental observations the model predicts that the testosterone concentration in the follicular fluid declines while the estradiol concentration increases as the follicle grows and develops (Figure 5E,F). Overall, the receptor patterns emerging in the simulation closely match the observed expression patterns. This demonstrates that the observed expression patterns directly result from the reported regulatory interactions without need for further restrictions. Interestingly, MAPK-signalling has been shown to be required for the spatial propagation of LH-dependent signalling [130]. This is in good agreement with the model which requires IGF-dependentsignalling for the emergence of LH-receptors in the granulosa.

We obtained these consistent results without requiring different signalling impacts of FSH and LH. Their differential impact in the model results entirely from the different expression patterns of their respective receptors, protein concentrations, binding affinities, diffusion constants, and signalling thresholds. It has previously been noticed that their intracellular signalling responses are virtually identical [65-69]. A similar case of where two homologous proteins take different roles even though they connect to the same signalling machinery has been reported for FGF4 and FGF8 during limb bud development. FGF4 and FGF8 have different roles, yet if the *Fgf4* gene is expressed from the *Fgf8*-regulatory sequence instead of *Fgf8* it can take over the function of FGF8 [131]. It is thus the differential spatio-temporal expression control rather than the differences on the protein level that convey the different functions.

With a data-based, validated model at hand it should now become feasible to investigate the molecular basis of infertility with a more integrated approach that reflects the tight coupling between the many regulatory processes. Most cases of primary ovarian insufficiency have remained unexplained so far. Future work should also focus on understanding species-specific differences. The model was mainly built with data from cattle, whereas all knock-out data originate from transgenic mouse models. Murine follicles differ from bovine and human follicles in rapid maturation and small size with a diameter of only 0.5-0.6 mm at ovulation [1]. Given the small size of the murine follicles the diffusional gradients must be steeper to scale with the size of the domain. Here it is interesting to note that the affinity of human LH has been found to be about 100-fold higher to rat LH-receptor than to human LH-receptor [37].

To further apply the model to human ovarian follicle development and infertility it will be important to establish all key parameters in human follicles as well and to develop a 3D-model. The diameters as well as the follicular fluid volume and granulosa cell numbers of growing human follicles have been reported previously [13,14] and further measurements can now be made using the latest imaging technology, such as magnetic resonance imaging (MRI). Moreover, it is now increasingly feasible to obtain gene expression kinetics from *in vivo* and cultured follicles at defined stages and to manipulate *in vitro* cultured follicles. It will then be interesting to address the detailed impacts of changes in binding, decay, and production rates as decay rates and binding affinities are known to vary not only with age but also during the follicular phase of the menstrual cycle due to changes in the glycosylic moiety of the hormone [56,132]. In patients undergoing assisted reproduction the feedback with the pituitary is voluntarily disrupted. When active, this feedback leads to changes in the serum levels of FSH and LH in response to changes in estradiol secretion. To better understand the manifestation of disease it may become important to also include this feedback in the model rather than constant concentrations of FSH and LH.

Data-based, validated computational models of biomedical processes are still rare, but they are likely to become invaluable tools to define the molecular causes of disease and to develop novel therapeutic approaches that respect the complex regulatory logic of biological systems.

## Competing interests

The authors declare that they have no competing interests.

## Authors’ contributions

DI developed the model. DI and CDG wrote the paper. Both authors read and approved the final manuscript.

## Supplementary Material

Additional file 1 **Figure S1 Time-dependent expression profiles in the follicle.** This figure is the same as Figure 3, except that the thickness of the thecal layer is increased by 20%. Simulated and measured expression levels of LH receptor in granulosa and theca, and of the FSH receptor and the aromatase in the granulosa during the 1st wave of the bovine follicle maturation process. The data was recorded by [15,16]. The lower curves that extend to 10 days are the measurements in [16]; the follicles in these measurements were growing more slowly than in the study by [15]. Note that the dominant follicle undergoes atresia from day 6. **(A)** Data (dotted lines) and simulation predictions (solid line) of LH receptor expression in the theca. **(B)** Data (dotted lines) and simulation predictions (solid line) of LH receptor expression in the granulosa. **(C)** Data (dotted lines) and simulation predictions (solid line) of FSH receptor expression in the granulosa. **(D)** Data (dotted lines) and simulation predictions (solid line) of aromatase expression in the granulosa. Click here for file

Additional file 2 **Figure S2 FSH and LH gradient formation in the follicle.** This figure is the same as Figure 4, except that the thickness of the thecal layer is increased by 20%. At time zero no hormones are present in the follicle (black line). Over the next five days LH and FSH diffuse into the follicular domain from the boundary (theca) and form a gradient. The five time points are equally spaced at 0 (black), 1 (cyan), 2 (blue), 3 (green), 4 (yellow), and 5 days (red) curves. Panels A and B show the profiles on the growing domain. Panels C and D show the concentration profiles on a scaled domain. Panels E and F show the concentration profiles on a scaled domain if we include the fluid-filled antrum on one site of the COC block. Panels G and H show the concentration profiles on a scaled domain if we assume rapid mixing in the fluid-filled antrum. The shading indicates the different parts of the follicle, i.e. theca (white), granulosa cells (dark grey), cumulus cells (light grey), and follicular fluid (blue). Note that the theca and granulosa layers are very thin and thus barely visible. Click here for file

Additional file 3 **Figure S3 The production of androgens and estradiol in the follicle.** This figure is the same as Figure 5, except that the thickness of the thecal layer is increased by 20%. (A-B) Production of (A) androgens and (B) estradiol. Only part of the domain is shown, i.e. theca (white), granulosa cells (dark grey), and a part of the COC (light grey). (C-D) Concentration profiles of (C) androgens and (D) estradiol. The five time points are equally spaced at 0 (black), 1 (cyan), 2 (blue), 3 (green), 4 (yellow), and 5 days (red) curves. All panels show the concentration profiles on a scaled domain. The shading indicates the different parts of the follicle, i.e. theca (white), granulosa cells (dark grey), COC (light grey), and follicular fluid (blue). Androgens are produced only in the theca, and estradiol is produced only in the granulosa cells. In the follicular fluid steroids are neither produced nor degraded. (E-F) The average steroid concentrations of (E) androgens and (F) estradiol in the follicular fluid over time. Click here for file

Additional file 4 **Figure S4 The emergence of FSH-, LH-, and estrogen receptor gradients in the follicle.** This figure is the same as Figure 6, except that the thickness of the thecal layer is increased by 20%. (A-C) Receptor profiles of (A) FSH-receptor, (B) LH-receptor, (C) estrogen receptor. At time zero the receptor concentrations are low and the receptors are distributed homogenously in the follicle (black line). Over the next five days localised feedbacks create a graded distribution of the receptors. (D-E) Receptor production. (F-H) Unbound receptors (I-K) Bound receptors (L-N) Receptor activity. The five time points are equally spaced at 0 (black), 1 (cyan), 2 (blue), 3 (green), 4 (yellow), and 5 days (red) curves. All panels show the concentration profiles on a scaled domain. For better readability we only show the subset of the domain that includes the theca (white area), granulosa (dark shade), COC (light shade), and part of the follicular fluid (blue shade) on the right hand side of the domain. Click here for file
