# Supplementary figures and images for: Computational modelling of bovine ovarian follicle development
Source: BMC Syst Biol. 2013 Jul 15;7:60. doi: 10.1186/1752-0509-7-60 (PMC3726369; doi:10.1186/1752-0509-7-60)

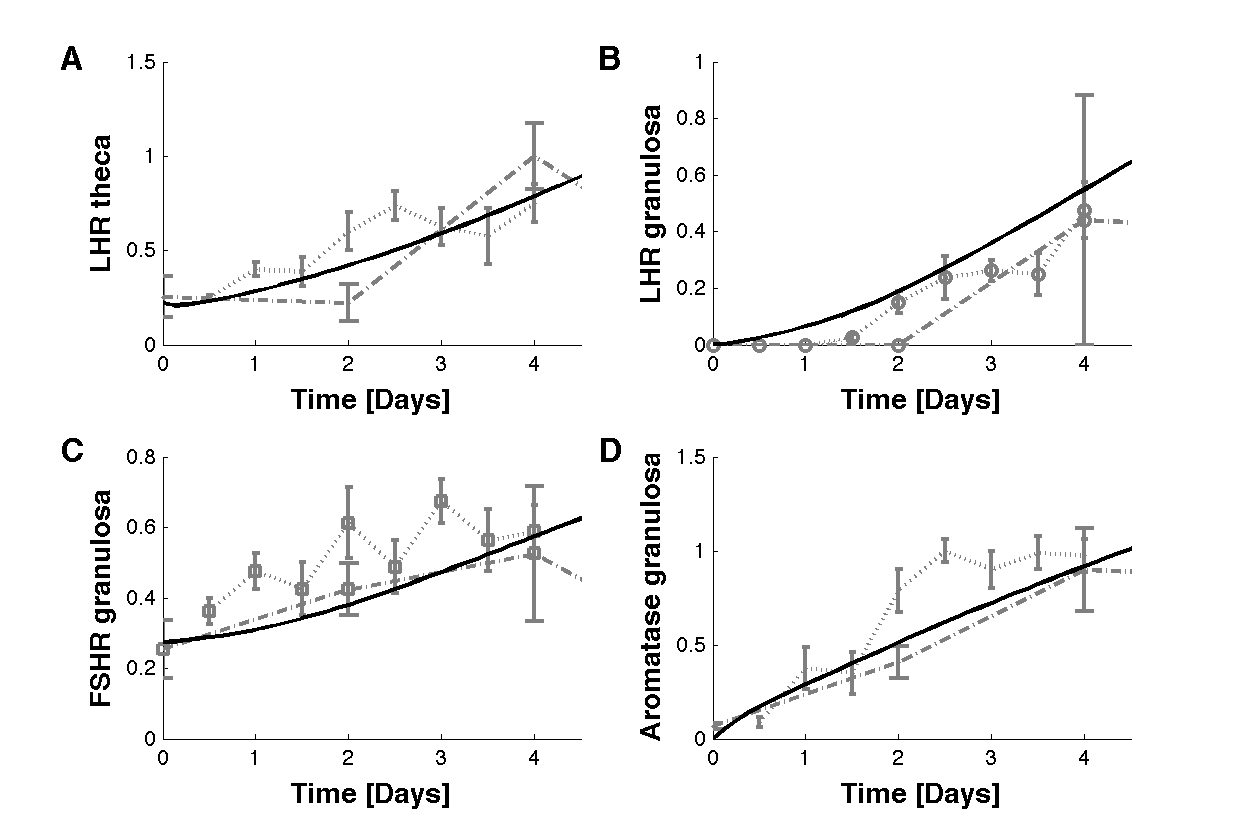

Supplement: Additional file 1 — Figure S1 Time-dependent expression profiles in the follicle. This figure is the same as Figure 3, except that the thickness of the thecal layer is increased by 20%. Simulated and measured expression levels of LH receptor in granulosa and theca, and of the FSH receptor and the aromatase in the granulosa during the 1st wave of the bovine follicle maturation process. The data was recorded by [15,16]. The lower curves that extend to 10 days are the measurements in [16]; the follicles in these measurements were growing more slowly than in the study by [15]. Note that the dominant follicle undergoes atresia from day 6. (A) Data (dotted lines) and simulation predictions (solid line) of LH receptor expression in the theca. (B) Data (dotted lines) and simulation predictions (solid line) of LH receptor expression in the granulosa. (C) Data (dotted lines) and simulation predictions (solid line) of FSH receptor expression in the granulosa. (D) Data (dotted lines) and simulation predictions (solid line) of aromatase expression in the granulosa. [file 1752-0509-7-60-S1.png]

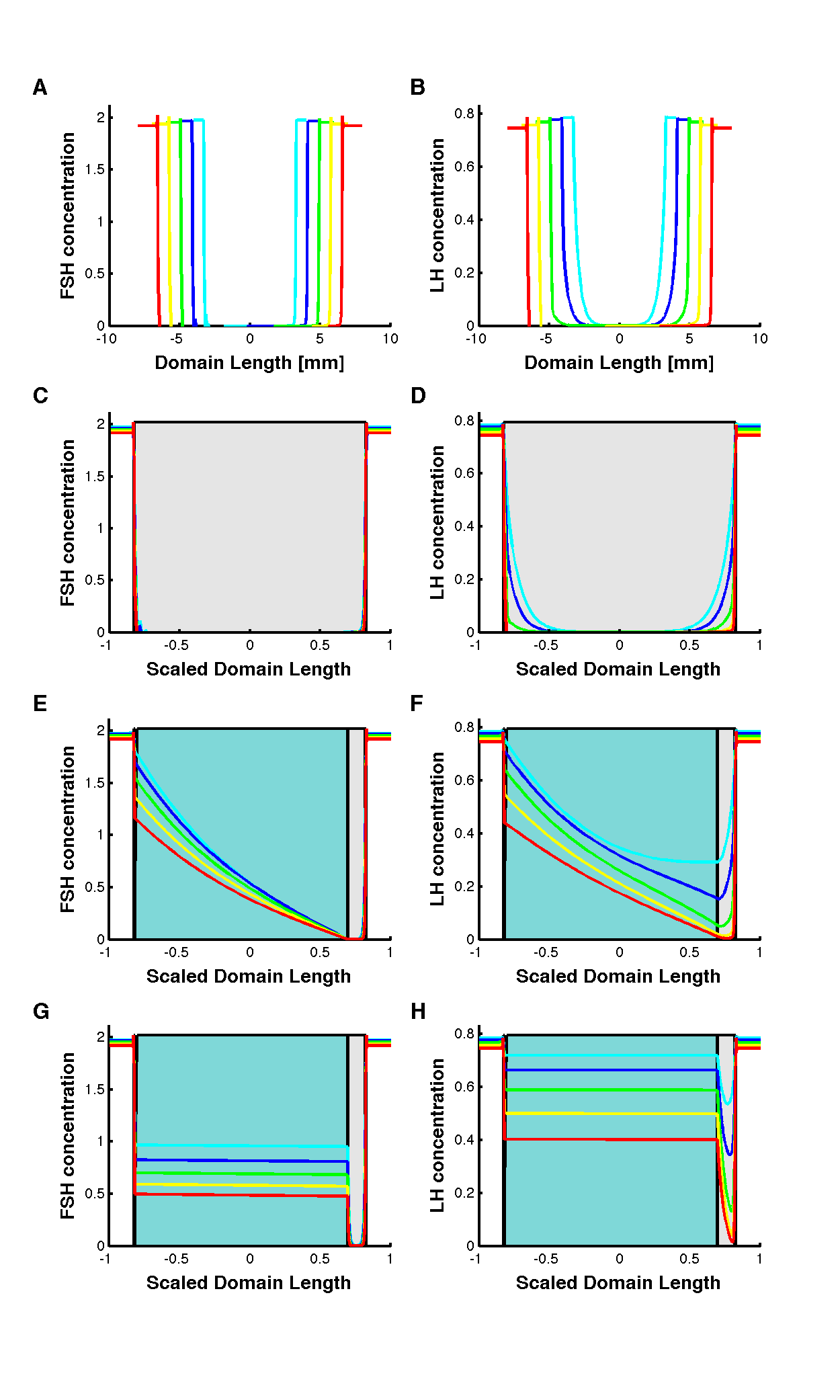

Supplement: Additional file 2 — Figure S2 FSH and LH gradient formation in the follicle. This figure is the same as Figure 4, except that the thickness of the thecal layer is increased by 20%. At time zero no hormones are present in the follicle (black line). Over the next five days LH and FSH diffuse into the follicular domain from the boundary (theca) and form a gradient. The five time points are equally spaced at 0 (black), 1 (cyan), 2 (blue), 3 (green), 4 (yellow), and 5 days (red) curves. Panels A and B show the profiles on the growing domain. Panels C and D show the concentration profiles on a scaled domain. Panels E and F show the concentration profiles on a scaled domain if we include the fluid-filled antrum on one site of the COC block. Panels G and H show the concentration profiles on a scaled domain if we assume rapid mixing in the fluid-filled antrum. The shading indicates the different parts of the follicle, i.e. theca (white), granulosa cells (dark grey), cumulus cells (light grey), and follicular fluid (blue). Note that the theca and granulosa layers are very thin and thus barely visible. [file 1752-0509-7-60-S2.png]

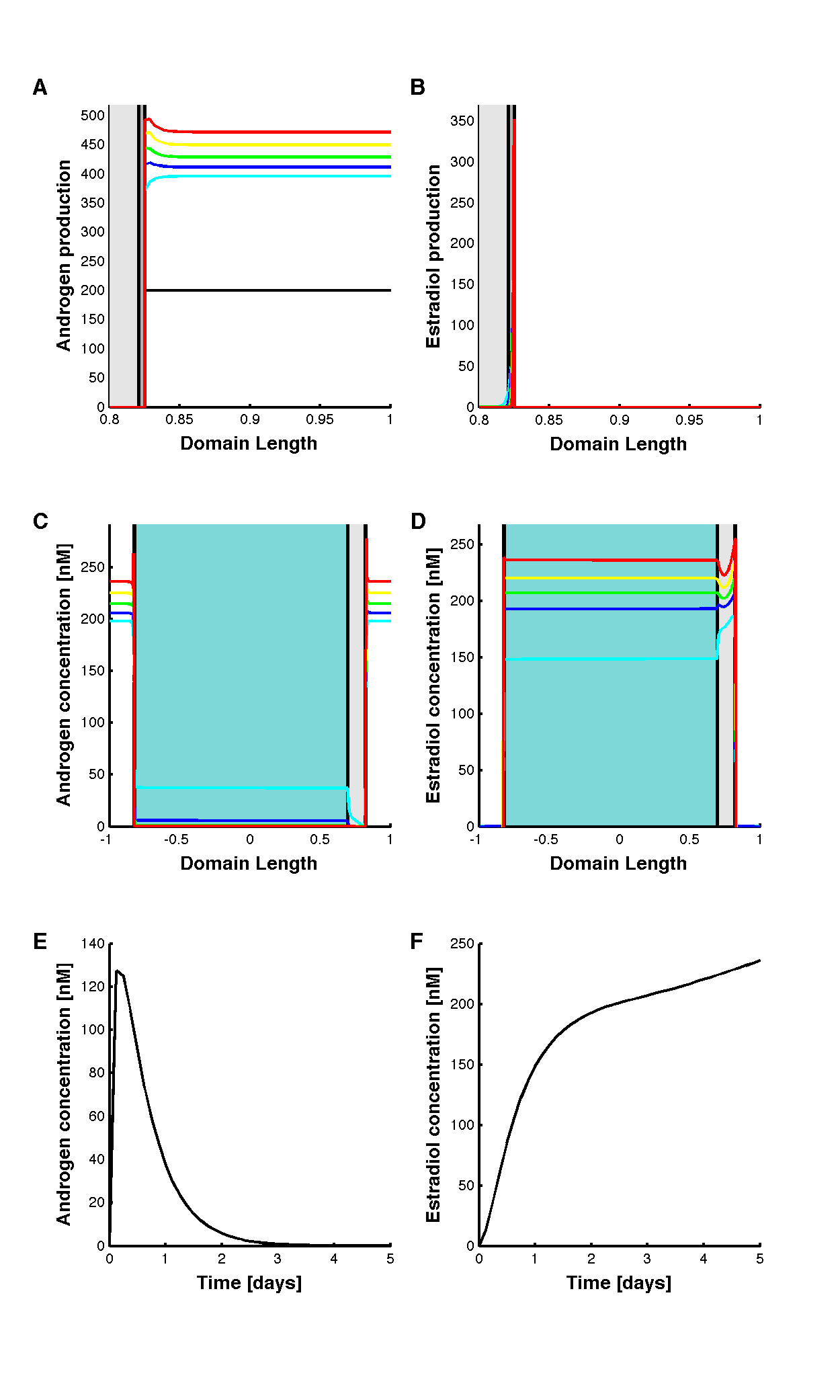

Supplement: Additional file 3 — Figure S3 The production of androgens and estradiol in the follicle. This figure is the same as Figure 5, except that the thickness of the thecal layer is increased by 20%. (A-B) Production of (A) androgens and (B) estradiol. Only part of the domain is shown, i.e. theca (white), granulosa cells (dark grey), and a part of the COC (light grey). (C-D) Concentration profiles of (C) androgens and (D) estradiol. The five time points are equally spaced at 0 (black), 1 (cyan), 2 (blue), 3 (green), 4 (yellow), and 5 days (red) curves. All panels show the concentration profiles on a scaled domain. The shading indicates the different parts of the follicle, i.e. theca (white), granulosa cells (dark grey), COC (light grey), and follicular fluid (blue). Androgens are produced only in the theca, and estradiol is produced only in the granulosa cells. In the follicular fluid steroids are neither produced nor degraded. (E-F) The average steroid concentrations of (E) androgens and (F) estradiol in the follicular fluid over time. [file 1752-0509-7-60-S3.png]

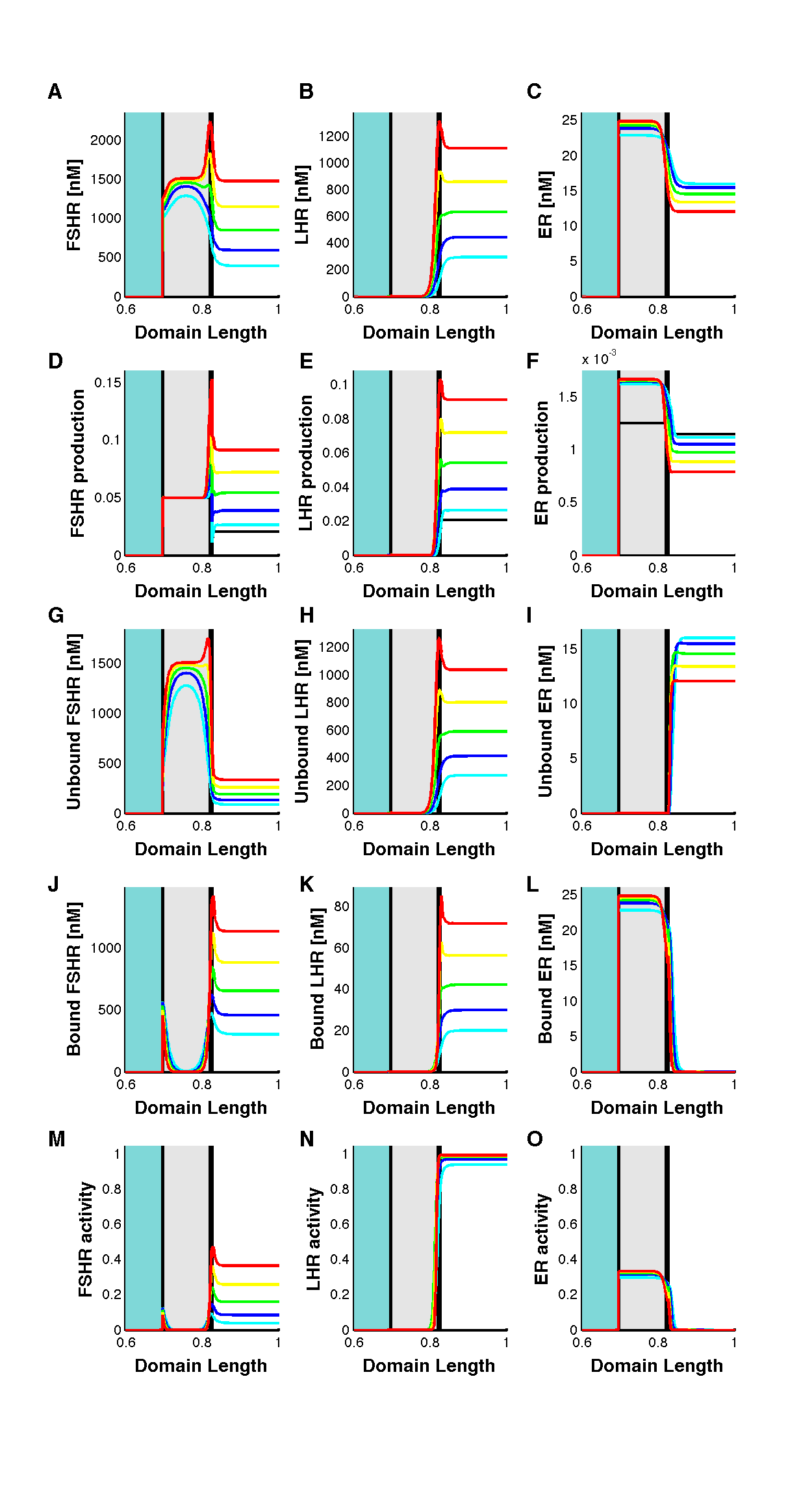

Supplement: Additional file 4 — Figure S4 The emergence of FSH-, LH-, and estrogen receptor gradients in the follicle. This figure is the same as Figure 6, except that the thickness of the thecal layer is increased by 20%. (A-C) Receptor profiles of (A) FSH-receptor, (B) LH-receptor, (C) estrogen receptor. At time zero the receptor concentrations are low and the receptors are distributed homogenously in the follicle (black line). Over the next five days localised feedbacks create a graded distribution of the receptors. (D-E) Receptor production. (F-H) Unbound receptors (I-K) Bound receptors (L-N) Receptor activity. The five time points are equally spaced at 0 (black), 1 (cyan), 2 (blue), 3 (green), 4 (yellow), and 5 days (red) curves. All panels show the concentration profiles on a scaled domain. For better readability we only show the subset of the domain that includes the theca (white area), granulosa (dark shade), COC (light shade), and part of the follicular fluid (blue shade) on the right hand side of the domain. [file 1752-0509-7-60-S4.png]
